# A *Yersinia* Effector with Enhanced Inhibitory Activity on the NF-κB Pathway Activates the NLRP3/ASC/Caspase-1 Inflammasome in Macrophages

**DOI:** 10.1371/journal.ppat.1002026

**Published:** 2011-04-21

**Authors:** Ying Zheng, Sarit Lilo, Igor E. Brodsky, Yue Zhang, Ruslan Medzhitov, Kenneth B. Marcu, James B. Bliska

**Affiliations:** 1 Department of Molecular Genetics and Microbiology, Center for Infectious Diseases, Stony Brook University, Stony Brook, New York, United States of America; 2 Section of Immunobiology, Yale University School of Medicine, New Haven, Connecticut, United States of America; 3 Department of Biochemistry and Cell Biology, Stony Brook University, Stony Brook, New York, United States of America; 4 Immunology and Genetics Department, Istituti Ortopedia Rizzoli, Bologna, Italy; ETH Zürich, Switzerland

## Abstract

A type III secretion system (T3SS) in pathogenic *Yersinia* species functions to translocate Yop effectors, which modulate cytokine production and regulate cell death in macrophages. Distinct pathways of T3SS-dependent cell death and caspase-1 activation occur in *Yersinia-*infected macrophages. One pathway of cell death and caspase-1 activation in macrophages requires the effector YopJ. YopJ is an acetyltransferase that inactivates MAPK kinases and IKKβ to cause TLR4-dependent apoptosis in naïve macrophages. A YopJ isoform in *Y. pestis* KIM (YopJ^KIM^) has two amino acid substitutions, F177L and K206E, not present in YopJ proteins of *Y. pseudotuberculosis* and *Y. pestis* CO92. As compared to other YopJ isoforms, YopJ^KIM^ causes increased apoptosis, caspase-1 activation, and secretion of IL-1β in *Yersinia-*infected macrophages. The molecular basis for increased apoptosis and activation of caspase-1 by YopJ^KIM^ in *Yersinia*-infected macrophages was studied. Site directed mutagenesis showed that the F177L and K206E substitutions in YopJ^KIM^ were important for enhanced apoptosis, caspase-1 activation, and IL-1β secretion. As compared to YopJ^CO92^, YopJ^KIM^ displayed an enhanced capacity to inhibit phosphorylation of IκB-α in macrophages and to bind IKKβ in vitro. YopJ^KIM^ also showed a moderately increased ability to inhibit phosphorylation of MAPKs. Increased caspase-1 cleavage and IL-1β secretion occurred in IKKβ-deficient macrophages infected with *Y. pestis* expressing YopJ^CO92^, confirming that the NF-κB pathway can negatively regulate inflammasome activation. K^+^ efflux, NLRP3 and ASC were important for secretion of IL-1β in response to *Y. pestis* KIM infection as shown using macrophages lacking inflammasome components or by the addition of exogenous KCl. These data show that caspase-1 is activated in naïve macrophages in response to infection with a pathogen that inhibits IKKβ and MAPK kinases and induces TLR4-dependent apoptosis. This pro-inflammatory form of apoptosis may represent an early innate immune response to highly virulent pathogens such as *Y. pestis* KIM that have evolved an enhanced ability to inhibit host signaling pathways.

## Author Summary

Pathogenic bacteria in the genus *Yersinia* use multiple virulence determinants to counteract innate immunity and facilitate infection. A type III system in *Yersinia* translocates an effector called YopJ that elicits cell death in macrophages. YopJ inhibits the production of survival factors in naïve macrophages, causing them to die by apoptosis, which is generally considered to be immunologically silent. However, recent studies show that caspase-1, a key regulator of pro-inflammatory responses, is activated in *Yersinia-*infected macrophages undergoing apoptosis. How caspase-1 is activated during YopJ-induced macrophage apoptosis is not known. We have identified a distinct isoform of YopJ in *Y. pestis* (YopJ^KIM^) that induces high levels of apoptosis and caspase-1 activation in infected macrophages. In this study, the molecular basis for the increased activity of YopJ^KIM^ was studied with the goal of better understanding the underlying mechanism of caspase-1 activation. The data show that YopJ^KIM^ has two amino acid changes that give it an enhanced ability to inhibit survival signals in macrophages. The increased apoptosis may cause membrane permeability, resulting in efflux of ions and activation of caspase-1. Therefore, apoptosis of naïve macrophages inflicted by highly virulent pathogens may not be immunologically silent.

## Introduction

Microbial pathogens encode numerous types of virulence factors that are used to circumvent or usurp immune responses within cells of their hosts. A protein export pathway known as the type III secretion system (T3SS) allows Gram-negative bacterial pathogens to deliver effector proteins into or across the plasma membrane of host cells, with the goal of co-opting or disrupting eukaryotic signaling pathways [1], [2]. Infection of macrophages with T3SS-expressing bacterial pathogens commonly causes cytotoxicity in the host cell, but the mechanisms of cellular demise and the morphological and immunological characteristics of cell death can be unique for each microbe [3]. Two types of macrophage death that can be induced by T3SS-expressing pathogens and distinguished morphologically and immunologically are apoptosis and pyroptosis [4]. Apoptosis is traditionally associated with a lack of inflammation while pyroptosis is considered pro-inflammatory [4], [5]. Apoptosis and pyroptosis can also be distinguished mechanistically by the fact that only the latter mechanism of cell death is dependent upon the activity of caspase-1, a pro-inflammatory caspase [4], [5]. Recently, however it has been determined that caspase-1 can be activated in macrophages dying of apoptosis [6], [7], [8], indicating that pathogen-inflicted apoptosis may not be immunologically silent.

Caspase-1 is synthesized as a 45 kDa inactive zymogen that is cleaved to generate the active heterotetramer composed of two p10 and two p20 subunits [9]. Activation of caspase-1 occurs through its recruitment to an inflammasome complex [10], [11], [12]. Activated caspase-1 cleaves pro-IL-1β and pro-IL-18, and promotes secretion of the mature forms of these cytokines by a non-conventional pathway. Macrophages dying of pyroptosis therefore release active forms of IL-1β, and IL-18, which are important cytokines for protective host responses against several pathogens [5]. In addition, pyroptosis can release intracellular bacteria from macrophages, allowing for clearance of the pathogens by neutrophils [13].

Inflammasome complexes assemble on a scaffold of NOD-like receptors **(**NLRs) [11], [12], [14]. NLRs comprise a family of pattern recognition receptors (PRRs) that detect cytosolic pathogen-associated molecular patterns (PAMPs) or infection-associated processes [10], [11], [12]. Well-studied NLR family members include NLRP3 (formerly NALP3 or Cryopyrin), NLRC4 (formerly IPAF) and NAIP5. NLRP3 in complex with the adaptor protein ASC induces the activation of caspase-1 in response to a variety of microbial products as well as endogenous danger signals such as potassium (K^+^) efflux or extracellular ATP [10], [11], [12]. NLRC4 recognizes bacterial flagellin from *S. enterica* serovar Typhimurium, which is delivered into macrophages via a T3SS in this pathogen [10], [13], [15], [16], [17]. Another family of PRRs, the toll-like receptors (TLRs) often function in concert with NLRs to positively regulate inflammasome activation and function [18]. For example, production of pro-IL-1β and pro-IL-18 is upregulated by TLR signaling. In addition, production of NLRP3 is positively regulated by TLR signaling through the NF-κB pathway [19].

In pathogenic *Yersinia* species, a plasmid-encoded T3SS delivers Yop effectors into host cells [2], allowing the bacteria to modulate innate immune responses [20]. The T3SS is an essential virulence determinant in these pathogens, which cause diseases ranging from plague (*Y. pestis*) to enterocolitis (*Y. enterocolitica*) and mesenteric lymphadenitis (*Y. pseudotuberculosis*). Naïve *Yersinia*-infected macrophages undergo apoptosis via a cell death program that requires TLR4-dependent activation of initiator and executioner caspases and T3SS-mediated delivery of YopJ, which inhibits expression of anti-apoptotic factors under regulatory control of MAPK and NF-κB signaling pathways [21], [22], [23], [24]. Inactivation of the NF-κB and MAPK signaling pathways via YopJ-mediated inhibition of the inhibitor of kappa B kinase beta (IKKβ) and MAPK kinases (MKKs) is critical for apoptosis of *Yersinia*-infected macrophages [23], [25].

YopJ is the prototypical member of a family of T3SS effectors that inhibit the NF-κB pathway [26], [27], [28]. These proteins exhibit homology to CE cysteine proteases [26]. Evidence has been obtained that YopJ can function as a deubiquitinase [29], [30], [31]. However, more recent studies indicate that YopJ has acetyltransferase activity, acetylating Ser and Thr residues critical for the activation of the MKKs and IKKβ [32], [33], [34]. YopJ is an important virulence factor in *Y. pseudotuberculosis* [21], [35] and *Y. enterocolitica,* where it is known as YopP [36].

Recently, it has been determined that caspase-1 can be activated in a T3SS-dependent manner by two distinct pathways in macrophages infected with *Yersinia* [6]. In one pathway, insertion of channels or pores in the plasma membrane by the T3SS translocon activates caspase-1 and causes pyroptosis in *Yersinia*-infected macrophages [6], [37], [38], [39]. Activation of caspase-1 in response to the *Yersinia* T3SS translocon can be counteracted by Yop effectors including YopE [39] and YopK [6], and therefore this pathway is inhibited in macrophages infected with wild-type bacteria.

A second pathway of caspase-1 activation that occurs in macrophages infected with wild-type *Yersinia* is not inhibited by YopE or YopK and requires YopJ activity [6], [8]. Although caspase-1 is activated in response to YopJ activity, caspase-1 is not required for YopJ-dependent macrophage apoptosis [6], [8]. A potential explanation for the ability of YopJ to cause caspase-1 activation came from the work of Greten et al. [7], who showed that genetic or pharmacological ablation of IKKβ resulted in apoptosis, activation of caspase-1 and secretion of IL-1β from macrophages following stimulation of TLR4 with LPS. Evidence was obtained that an anti-apoptosis gene product expressed under control of NF-κB, plasminogen activator inhibitor 2 (PAI-2), negatively regulates apoptosis and caspase-1 activation in LPS-stimulated macrophages [7]. The authors suggested that inhibition of caspase-1 by the NF-κB pathway represents a negative feedback loop that allows the innate immune system to activate, via TLR4, a compensatory host defense response against Gram-negative pathogens that inhibit activation of NF-κB [7].

Different *Yersinia* strains display a range of YopJ-dependent apoptotic activities on macrophages [8], [35], [40], [41]. This difference in apoptotic activity is due to the expression of distinct YopJ isoforms by different *Yersinia* strains [8], [35], [40], [41]. For example, a *Y. enterocolitica* strain encoding a YopP protein with an Arg at position 143 was shown to have higher apoptotic activity and inhibit IKKβ more efficiently in macrophages then strains having a Ser at this position [40]. *Y. pestis* KIM, a 2.MED (Mediaevalis) biovar strain, encodes a YopJ isoform that causes higher levels of apoptosis and caspase-1 activation in infected macrophages as compared to other YopJ isoforms [8]. The YopJ^KIM^ protein has an Arg at position 143 but in addition has two amino acid substitutions, at positions 177 and 206, as compared to other isoforms of this effector found in *Y. pestis* or *Y. pseudotuberculosis* strains.

Here, the molecular basis for the enhanced ability of YopJ^KIM^ to cause apoptosis and activate caspase-1 in *Yersinia*-infected macrophages was studied, with the goal of better understanding the underlying mechanism of this host response. Analysis of YopJ^KIM^ in parallel with other YopJ isoforms indicated that the unique capacity of this effector to cause high-level apoptosis and caspase-1 activation requires both codon substitutions at positions 177 and 206. The presence of these codon substitutions also correlated with the enhanced ability of YopJ^KIM^ to inhibit MKK and IKKβ signaling pathways. Infection of IKKβ-deficient macrophages with *Y. pestis* confirmed that this kinase has an important role in negatively regulating caspase-1 activation [7]. Finally, evidence was obtained that K^+^ efflux leading to activation of the NLRP3/ASC/capsase-1 inflammasome is important for secretion of IL-1β and IL-18 from macrophages infected with *Y. pestis* KIM. These findings indicate that, by inhibiting production of survival factors under control of the NF-κB and MAPK pathways, YopJ causes TLR4-dependent apoptosis and caspase-1 activation in macrophages infected with *Yersinia*. In addition, *Y. pestis* KIM causes high levels of apoptosis and caspase-1 activation in macrophages because it has evolved a YopJ isoform with enhanced inhibitory activity on NF-κB and MAPK pathways.

## Results

### Identification of amino acid substitutions in YopJ that increase MyD88- and Trif-dependent caspase-1 activation in *Yersinia*-infected macrophages

Sequence comparisons were made between YopJ^KIM^ and YopJ proteins from two other *Yersinia* strains that display lower apoptosis activity in macrophages, *Y. pseudotuberculosis* IP2666 and *Y. pestis* CO92. There is one amino acid difference between YopJ^KIM^ and YopJ in *Y. pseudotuberculosis* (YopJ^YPTB^), corresponding to L177F in the predicted catalytic core [29] of the enzyme (residues 109-194; Figure S1 in Text S1). Comparison of YopJ^KIM^ with YopJ from *Y. pestis* CO92 (YopJ^CO92^) revealed two differences, L177F and E206K, the latter of which is located just beyond the carboxy-terminal end of the predicted catalytic core (Figure S1 in Text S1).

To determine if the amino acid substitutions at positions 177 and 206 of YopJ^KIM^ affect secretion or delivery of the effector into macrophages, expression plasmids encoding YopJ^KIM^, YopJ^YPTB^, or YopJ^CO92^ appended with C-terminal GSK tags were constructed. An expression plasmid encoding a YopJ isoform with a Leu at position 177 and a Lys at position 206 (YopJ^KIME206K^) was also constructed in the same manner. The expression plasmids were introduced into a Δ*yopJ* mutant of *Y. pseudotuberculosis* (IP26; Table S1 in Text S1). *Y. pseudotuberculosis* was used in the experiment because it lacks the Pla protease of *Y. pestis* which is known to degrade Yops secreted in vitro [42]. The resulting strains were induced to secrete Yops under low calcium growth conditions and immunoblotting of the secreted proteins showed that YopJ^KIM^, YopJ^YPTB^, YopJ^KIME206K^ and YopJ^CO92^ were exported at equal levels (Figure S2 in Text S1).

**Figure 1:**
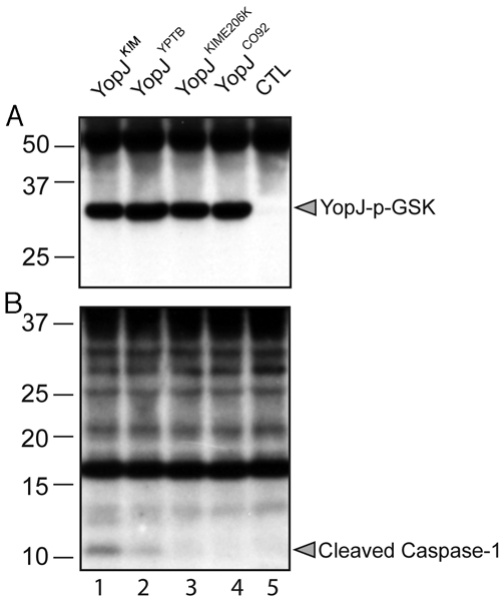
Translocation of different YopJ isoforms and caspase-1 activation in macrophages infected with *Y. pseudotuberculosis.* * Y. pseudotuberculosis* IP26 (IP2666Δ*yopJ)* carrying no pBAD plasmid as a control (CTL; lane 5) or pBAD vectors encoding the indicated YopJ-GSK isoforms (lanes 1–4), were grown under T3SS-inducing conditions in the presence of 0.2% of arabinose. Bacteria were added to BMDMs at an MOI of 20 and were allowed to infect for 2 hr. Arabinose (0.2%) was maintained in cell culture medium. Detergent lysates of infected macrophages were separated by SDS-PAGE and immunoblotting was performed with anti-phospho-GSK-3® antibody (A) and anti-caspase-1 antibody (B). Positions of molecular weight standards (kDa) are shown on the left and positions of YopJ- phospho-GSK and cleaved caspase-1 are shown on the right.

A translocation assay was performed using the phospho-GSK reporter system [43]. IP26 strains expressing the different YopJ isoforms fused to GSK were used to infect bone marrow derived macrophages (BMDMs) for 2 hr. Delivery of the effector into host cells was measured by anti-phospho-GSK immunoblotting [43]. The results showed that YopJ^KIM^, YopJ^YPTB^, YopJ^KIME206K^ and YopJ^CO92^ isoforms were translocated at similar levels (Figure 1A). Samples of the same lysates analyzed in Figure 1A were subjected to immunoblotting with anti-caspase-1 antibody to measure the level of caspase-1 cleavage. Consistent with previous results [6], cleavage of caspase-1 was detected in BMDMs infected with *Y. pseudotuberculosis* expressing YopJ^YPTB^ (Figure 1B, lane 2). However, caspase-1 cleavage was comparatively higher with expression of YopJ^KIM^ (lane 1) and lower with expression of YopJ^KIME206K^ or YopJ^CO92^ isoforms (lanes 3 and 4, respectively). These results suggest that the ability of YopJ^KIM^ to trigger maximal caspase-1 activation requires both the F177L and K206E substitutions, and these codon changes impart an activity to the protein that is manifested following its delivery into the host cell.

YopJ-mediated apoptosis in response to *Yersinia* infection requires stimulation of TLR4 in naïve macrophages to activate a death response pathway [25], [44]. It is not known if TLR signaling is required for YopJ-dependent activation of caspase-1 in *Yersinia-*infected macrophages. When BMDMs lacking the two major TLR adaptors, MyD88 and Trif, were infected with wild-type *Y. pseudotuberculosis* IP2666 for 2 hr, activation of caspase-1 was substantially reduced (Figure S3 in Text S1). Cleavage of caspase-1 was not diminished in IP2666-infected BMDMs missing only MyD88 or Trif (data not shown), indicating that TLR signaling through either of these adaptors is important for the downstream events that lead to activation of caspase-1 in conjunction with YopJ activity. YopJ-dependent caspase-1 activation and IL-1β secretion were inhibited when BMDMs were treated with LPS prior to infection with *Y. pseudotuberculosis* (Figure S3 in Text S1) [6] or *Y. pestis* [8]. Thus, macrophages pre-stimulated with LPS are desensitized to undergo YopJ-dependent apoptosis [38] and caspase-1 activation upon *Yersinia* infection. Desensitization occurs because the TLR4 signaling pathway contains a negative feed back mechanism operating via NF-κB that upregulates expression of proteins that inhibit apoptosis and activation of caspase-1 [7].

### The F177L and K206E substitutions in YopJ^KIM^ are important for increased apoptosis and secretion of IL-1β and IL-18 in *Y. pestis*-infected macrophages

**Figure 2:**
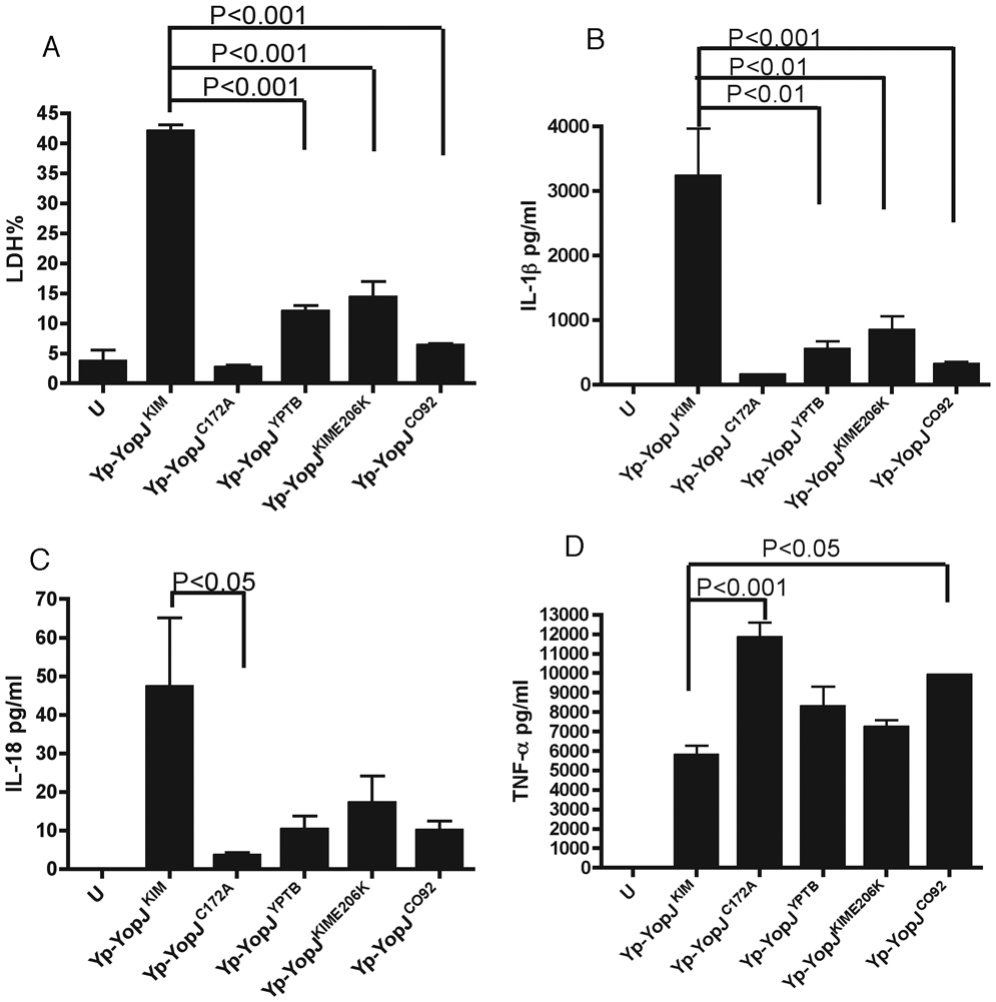
Cytokine secretion and cell death in macrophages infected with *Y. pestis* strains expressing different YopJ isoforms. BMDMs were left uninfected (U), or infected with the indicated Yp-YopJ strains at an MOI of 10. Supernatants collected after 24 hr of infection were used to measure cell death by LDH release (A) and secretion of IL-1β (B), IL-18 (C) and TNF-α (D) by ELISA. Results shown are the average of three independent experiments. Error bars represent standard deviation. Bracketing indicates P values (ANOVA) between different conditions.

To demonstrate that the polymorphisms in YopJ^KIM^ at positions 177 and 206 were important for the activity of this effector in the native context of *Y. pestis*, a L177F codon change was introduced into the sequence of *yopJ^KIM^* on the virulence plasmid pCD1 by allelic exchange, converting it to *yopJ^YPTB^*. In addition, an E206K codon change, a double L177F/E206K codon change, and a C172A codon change were introduced into pCD1, creating *yopJ^KIME206K^, yopJ^C092^*, and *yopJ^C172A^*, respectively. The resulting strains (referred to as Yp-YopJ^YPTB^, Yp-YopJ^KIME206K^, Yp-YopJ^C092^ and Yp-YopJ^C172A^)(Table S1 in Text S1) were phenotypically analyzed. As shown by immunoblotting of whole bacterial lysates, YopJ^KIM^, YopJ^YPTB^, YopJ^KIME206K^ and YopJ^CO92^ were expressed at equal levels in *Y. pestis* (Figure S4 in Text S1). The ability of *Y. pestis* strains expressing the different YopJ isoforms to induce apoptosis and cytokine secretion in BMDMs was then determined after a 24 hr infection. As shown in Figure 2A,B, the amounts of lactate dehydrogenase (LDH) released (used as a marker of cell death) and IL-1β secreted were significantly lower in macrophages infected with Yp-YopJ^YPTB^, Yp-YopJ^KIME206K^ or Yp-YopJ^CO92^ as compared to Yp-YopJ^KIM^. A similar trend was seen for secretion of IL-18 (Figure 2C).

Caspase-1 was required for the processing and release of IL-1β from macrophages under these infection conditions as shown by infecting wild-type or *casp-1* ^-/-^ BMDMs with Yp-YopJ^KIM^ and isolating IL-1β from infection supernatants by immunoprecipitation. Mature IL-1β was absent in supernatants isolated from *casp-1* ^-/-^ BMDMs infected with Yp-YopJ^KIM^ (Figure S5 in Text S1), indicating that the processing and release of IL-1β during infection of wild-type macrophages with Yp-YopJ^KIM^ occurred in a caspase-1-dependent manner.

As a control, levels of TNF-α, which is secreted independent of caspase-1 activity, were measured. Macrophages infected with Yp-YopJ^C172A^ or Yp-YopJ^CO92^ secreted significantly higher levels of TNF-α as compared to Yp-YopJ^KIM^, whereas the other mutants tested produced intermediate results (Figure 2D). Overall, these results indicate that amino acid substitutions at positions 177 and 206 are important for the ability of YopJ^KIM^ to induce high levels of macrophage apoptosis, caspase-1 activation and secretion of mature IL-1β and IL-18 in *Y. pestis*-infected macrophages. Conversely, the amino acid substitutions at positions 177 and 206 are important for the ability of YopJ^KIM^ to inhibit TNF-α secretion in macrophages under the same conditions.

### YopJ^KIM^ binds to IKKβ with higher affinity and more efficiently inhibits phosphorylation of IκBα as compared to YopJ^CO92^

**Figure 3:**
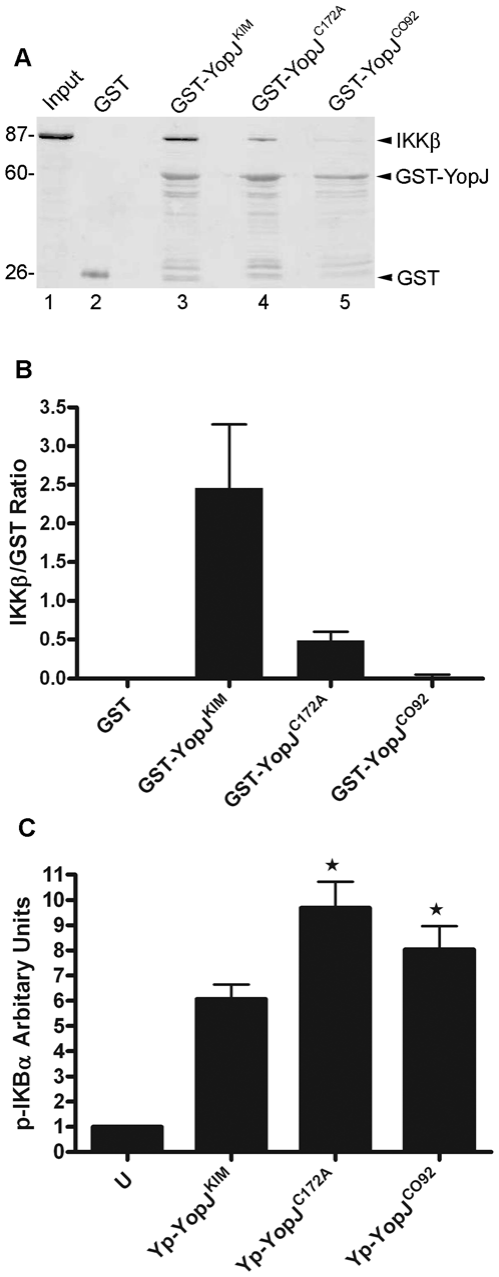
Measurement of IKKβ binding to different YopJ isoforms and phospho-IκBα levels in macrophages infected with different *Y. pestis* strains. (A) Binding of IKKβ to different YopJ isoforms as determined using a GST pull down procedure and lysates of transfected HEK293T cells. Purified proteins corresponding to GST (lane 2) or the indicated GST-YopJ fusion proteins (lanes 3–5) were immobilized on beads and incubated in cell lysates containing overexpressed IKKβ. After washing, proteins bound to the beads were detected and the signals quantified by immunoblotting using antibodies to IKKβ or GST and an Odyssey imaging system. Lane 1 contains a sample of the input transfected cell lysate (Input). Positions of molecular weight standards (kDa) are shown on the left and positions of IKKβ, GST-YopJ, and GST proteins are shown on the right. (B) Ratios of the signals for IKKβ and GST obtained by immunoblotting are presented in bar graph format, with values representing averages of two independent experiments. (C) BMDMs were left uninfected (U) or infected with Yp-YopJ^KIM^, Yp-YopJ^C172A^ or Yp-YopJ^CO92^ at an MOI of 50. At 1 hr post infection lysates of the infected macrophages were prepared and subjected to ELISA to determine levels of phospho (p)-IκBα. Results show p-IκBα values normalized to arbitrary units by setting uninfected to 1. Results were averaged from six (uninfected and Yp-YopJ^KIM^) or three (Yp-YopJ^C172A^ and Yp-YopJ^CO92^) independent experiments and error bars represent standard deviations. P value<0.05 (t test) as compared to Yp-YopJ^KIM^ condition is indicated by (*).

To determine if YopJ^KIM^ has higher affinity for IKKβ as compared to other YopJ isoforms, several different YopJ proteins were assayed for the ability to bind this kinase in cell lysates. Purified GST-YopJ fusion proteins or GST alone bound to beads were incubated in HEK293T cell lysates that contained overexpressed IKKβ. The amounts of IKKβ and GST proteins recovered on the beads after washing was measured by quantitative immunoblotting. IKKβ bound to beads coated with GST-YopJ^KIM^ but not to beads coated with GST alone (Figure 3A, compare lanes 2 and 3). There was reduced binding of IKKβ to GST-YopJ^CO92^ as compared to GST-YopJ^KIM^ (Figure 3A, compare lanes 3 and 5). When the amount of bound IKKβ was normalized to the amount of GST fusion protein recovered, it was estimated that 10-times less IKKβ bound to GST-YopJ^CO92^ as compared to GST-YopJ^KIM^ (Figure 3B). A GST fusion protein encoding YopJ^C172A^ bound ∼5 times less IKKβ as compared to GST-YopJ^KIM^ (Figure 3A, compare lanes 3 and 4, Figure 3B), suggesting that the catalytic Cys residue contributes to binding between IKKβ and YopJ^KIM^. Overall, these results suggest that YopJ^KIM^ has higher affinity for IKKβ as compared to YopJ^CO92^.

To determine if YopJ^KIM^ is a better inhibitor of IKKβ than YopJ^CO92^, the amount of phosphorylated IκBα (p-IκBα) in BMDMs was measured after a 1 hr infection. As shown in Figure 3C, significantly lower levels of p-IκBα were present in macrophages infected with Yp-YopJ^KIM^ as compared to BMDMs infected with Yp-YopJ^CO92^. In addition, significantly lower levels of p-IκBα were present in macrophages infected with Yp-YopJ^KIM^ as compared to BMDMs infected with Yp-YopJ^C172A^ (Fig. 3C), confirming that acetyltransferase activity is important for YopJ to inhibit the NF-κB pathway. Because IκBα is directly phosphorylated by IKKβ, these results are consistent with the idea that YopJ^KIM^ more efficiently inhibits IKKβ activity as compared to YopJ^CO92^.

### Partial genetic ablation of IKKβ increases caspase-1 activation in *Y. pestis-*infected macrophages

**Figure 4:**
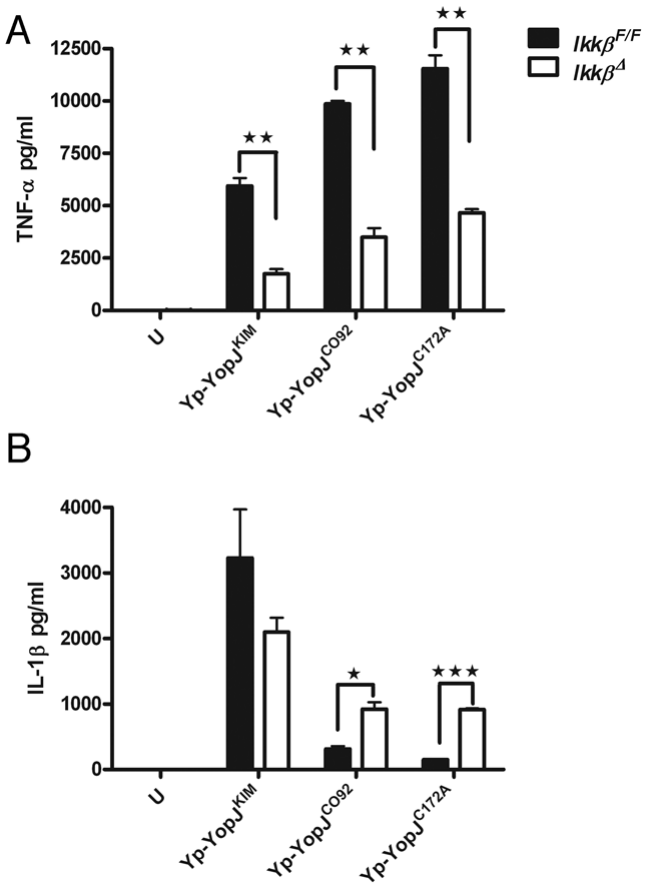
IL-1β and TNF-α secretion in *Ikk*β^F/F^ or *Ikk*β^Δ^ macrophages infected with *Y. pestis* strains expressing different YopJ isoforms. *Ikk*β^F/F^ or *Ikk*β**^Δ^** macrophages were left uninfected (U) or infected with the indicated Yp-YopJ strains at an MOI of 10. Twenty-four hr post infection, cell supernatants were collected. Secreted TNF-α (A) and IL-1β (B) were measured by ELISA. Results were averaged from three independent experiments, and error bars represent standard deviation. P values (t test) are indicated by bracketing (P<0.05 (*), P<0.01 (**), P<0.001(***).

Greten et al. have shown that treatment of IKKβ-deficient macrophages with LPS causes activation of caspase-1 and secretion of IL-1β [7]. If IKKβ activity is important to suppress activation of the inflammasome in macrophages infected with a live Gram-negative pathogen, than increased caspase-1 activation and IL-1β secretion should be observed in IKKβ-deficient as compared to wild-type BMDMs infected with *Y. pestis.* The effect of genetic inactivation of *Ikk*β on caspase-1 activation in *Y. pestis*-infected macrophages was therefore investigated. IKKβ-deficient BMDMs were generated by conditional Cre-lox-mediated deletion of a “floxed” *Ikk*β gene (referred to as *Ikk*β^Δ^ BMDMs; Materials and Methods). The *Ikk*β^Δ^ BMDMs or wild-type control *Ikk*β^F/F^ macrophages were left uninfected or infected with Yp*-*YopJ^KIM^, Yp*-*YopJ^CO92^ or Yp*-*YopJ^C172A^ for 4 hr. Quantitative RT-PCR (qRT-PCR) of *Ikk*β message was used to estimate the efficiency of Cre-lox mediated deletion of the *Ikk*β gene in the BMDMs. Results indicated that ∼50% of the *Ikk*β genes had been deleted in the population of *Ikk*β^Δ^ cells (Figure S6A in Text S1). The impact of this partial deficiency in *Ikk*β on the expression and secretion of cytokines in the *Y. pestis* infected macrophages was determined. As compared to the *Ikk*β^F/F^ macrophages, the *Ikk*β^Δ^ BMDMs were compromised for infection-induced expression of mRNA for the cytokines IL-18, TNFα and IL-1β, as shown by qRT-PCR (Figure S6B–D in Text S1). This result was expected since the NF-κB pathway positively regulates expression the il-18, tnf and il-1b genes. Accordingly, the *Ikk*β^Δ^ BMDMs secreted lower levels of TNFα as compared to *Ikk*β^F/F^ macrophages after a 24 hr infection (Figure 4A). In addition, during infection with Yp-YopJ^CO92^ or Yp-YopJ^C172A^, higher amounts of IL-1β were secreted from *Ikk*β^Δ^ BMDMs as compared to *Ikk*β^F/F^ macrophages (Figure 4B), consistent with the idea that the NF-κB pathway negatively regulates processing and secretion of IL-1β via control of caspase-1 activation [7]. Unexpectedly, the amount of IL-1β secreted following infection with Yp-YopJ^KIM^ appeared to be lower in *Ikk*β^Δ^ BMDMs as compared to *Ikk*β^F/F^ macrophages, although the observed difference was not statistically significant (Figure 4B). The interpretation of this latter result was complicated because of the fact that there was only partial deficiency in *Ikk*β in the *Ikk*β^Δ^ BMDMs, but one possible explanation was that synthesis of pro-IL-1β was reduced due to the extremely low level il-1b message in the *Ikk*β^Δ^ BMDMs infected with Yp-YopJ^KIM^ (Figure S6D in Text S1).

**Figure 5:**
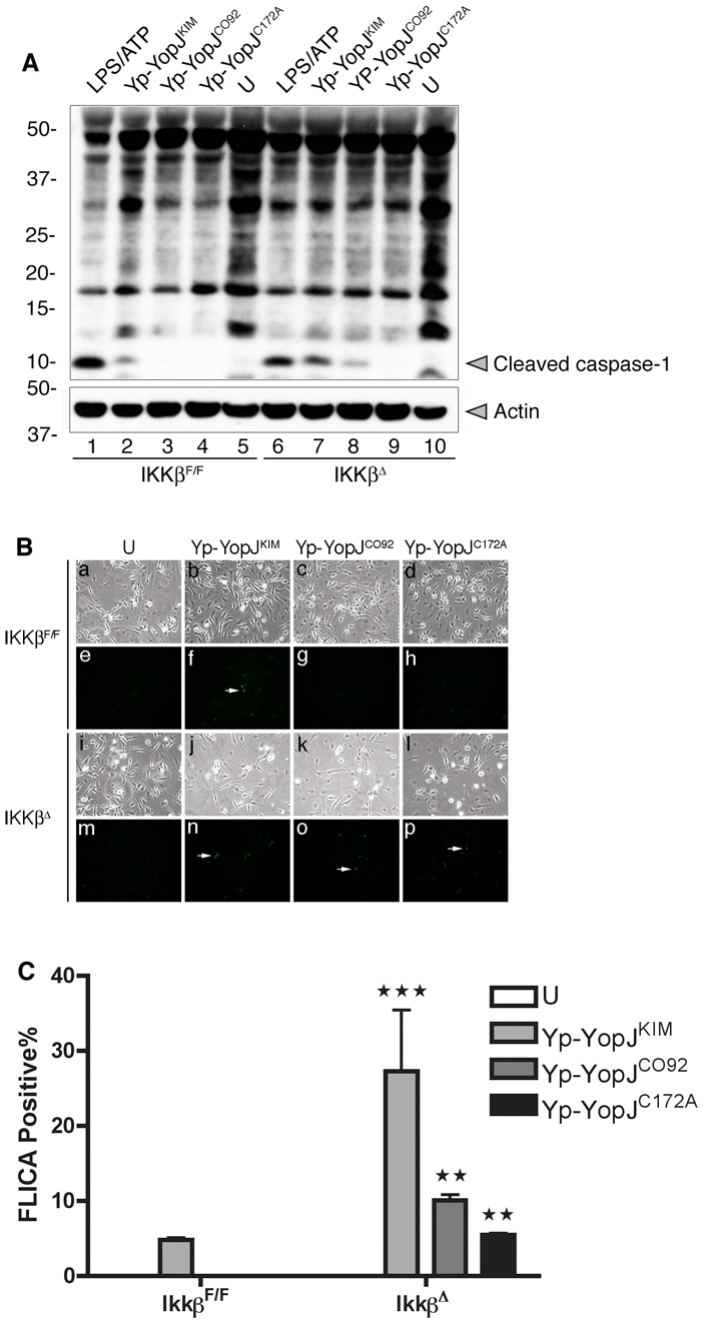
Caspase-1 activation in *Ikk*β^F/F^ or *Ikk*β^Δ^ macrophages infected with *Y. pestis* strains expressing different YopJ isoforms. *Ikk*β^F/F^ or *Ikk*β**^Δ^** BMDMs were left uninfected (U) or infected with the indicated Yp-YopJ strains at an MOI of 20 (A) or 10 (B and C), or treated with LPS for 3 hr and then exposed to ATP (LPS/ATP). (A) Caspase-1 cleavage was determined at 1 hr post ATP treatment or 2 hr post infection. In (A) samples of detergent lysates were separated by SDS-PAGE and immunoblotted with anti-caspase-1 antibody (upper panel) or anti-actin antibody antibody (lower panel). Positions of molecular weight standards (kDa) are shown on the left and positions of cleaved caspase-1 and actin are shown on the right. In (B) uninfected or infected macrophages on coverslips were incubated with FLICA reagent (FAM-YVAD-FMK) at 9 hr post infection to stain for active caspase-1 (green fluorescence). The samples were fixed, mounted on slides, and light microscopy was used to detect phase (a–d, i–l) or fluorescence (e-h, m-p) signals. Representative images of uninfected or infected cells were captured by digital photomicroscopy. White arrows point to FLICA positive cells. In (C), average percentages (error bars show standard deviation) of FLICA positive cells counted from three random fields per coverslip in three independent experiments is shown. P values comparing results of infection in *Ikk*β**^Δ^** to *Ikk*β^F/F^ BMDMs was determined (P<0.01, **; P<0.001, ***).

Activation of caspase-1 was measured by immunoblotting to detect the cleaved enzyme in lysates prepared 2 hr after infection of *Ikk*β^Δ^ or *Ikk*β^F/F^ BMDMs with Yp-YopJ^KIM^, Yp-YopJ^CO92^ or Yp-YopJ^C172A^. Caspase-1 activation in uninfected BMDMs or in macrophages treated with LPS and ATP was determined in parallel for comparison. Increased caspase-1 cleavage occured in *Ikk*β^Δ^ macrophages infected with Yp-YopJ^KIM^ or Yp-YopJ^CO92^ as compared to *Ikk*β^F/F^ BMDMs infected with the same strains (Figure 5A, compare lanes 7 and 8 with 2 and 3). Cleaved caspase-1 was below the limit of detection in *Ikk*β^Δ^ macrophages infected with Yp-YopJ^C172A^ (Figure 5A, lane 9). Activation of caspase-1 was also measured by a microscopic assay utilizing FAM-YVAD-FMK, a fluorescent probe for active caspase-1, in *Ikk*β^Δ^ or *Ikk*β^F/F^ BMDMs infected for 9 hr. The results showed overall higher levels of caspase-1 positive cells in *Ikk*β^Δ^ as compared to *Ikk*β^F/F^ macrophages (Figure 5B and C). Taken together, these results show that loss of IKKβ activity can increase caspase-1 activation in macrophages infected with *Y. pestis,* and are consistent with the idea that IKKβ is an important target of YopJ for activation of the inflammasome.

### YopJ^KIM^ more efficiently inhibits activation of MAPKs as compared to YopJ^CO92^

**Figure 6:**
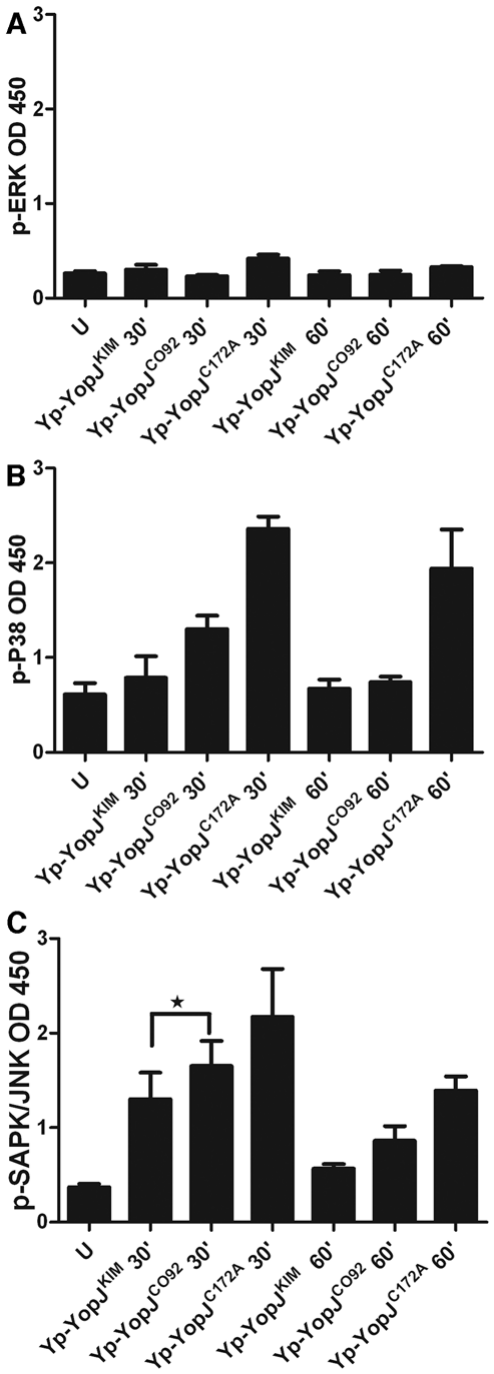
Measurement of phospho-MAPK levels in macrophages infected with *Y. pestis* strains expressing different YopJ isoforms. BMDMs were left uninfected (U), or infected with the indicated Yp-YopJ strains at an MOI of 20. At 30 or 60 min post infection lysates of the infected macrophages were prepared and subjected to ELISA to determine levels of phospho-ERK (A), -p38 (B) or –SAPK/JNK (C). Results show OD450 values averaged from three (uninfected, Yp-YopJ^KIM^ and Yp-YopJ^CO92^) or two (Yp-YopJ^C172A^) independent experiments and error bars represent standard deviations. P value (*, <0.05) (t test) is indicated by bracket.

In addition to binding to and acetylating IKKβ, YopJ binds to and acetylates other members of the MKK superfamily including MKK1, MKK2, MKK3, MKK4, MKK5, and MKK6 [26], [32], [33]. There is evidence that YopJ binds to a site conserved on members of the MKK-IKK superfamily [45]. Since we had previously obtained evidence that inhibition of MAPK signaling was critical for YopJ-induced macrophage apoptosis [23], we sought to determine if YopJ^KIM^ could more efficiently inhibit MAPK phosphorylation as compared to YopJ^CO92^. BMDMs were left uninfected or infected for 30 or 60 min with Yp*-*YopJ^KIM^, Yp-YopJ^CO92^, or Yp*-*YopJ^C172A^ and ELISA was used to measure phosphorylation of the MAPKs ERK (substrate of MKK1/2), p38 (substrate of MKK3/6) and SAPK/JNK (substrate of MKK4/7) (Materials and Methods). As shown in Figure 6A, ERK was not phosphorylated to a large degree at either time point in macrophages infected with Yp*-*YopJ^C172A^ and therefore it was not possible to evaluate the degree to which ERK phosphorylation was inhibited by either YopJ^KIM^ or Yp-YopJ^CO92^. In contrast, p38 and JNK did show increased phosphorylation upon infection with Yp*-*YopJ^C172A^, especially at the 30 min time point (Figure 6B and C, respectively). There was in general reduced phosphorylation of p38 and JNK in BMDMs infected with Yp-YopJ^KIM^ as compared to YopJ^CO92^, especially at the 30 min time point, and the difference was statistically significant in the case of JNK (Figure 6B and C). These results suggest that YopJ^KIM^ more efficiently inhibits the activities of MKK3/6 and MKK4/7 as compared to YopJ^CO92^.

### The NLRP3/ASC/caspase-1 inflammasome is important for secretion of IL-1β and IL-18 from macrophages infected with Yp-YopJ^KIM^

**Figure 7:**
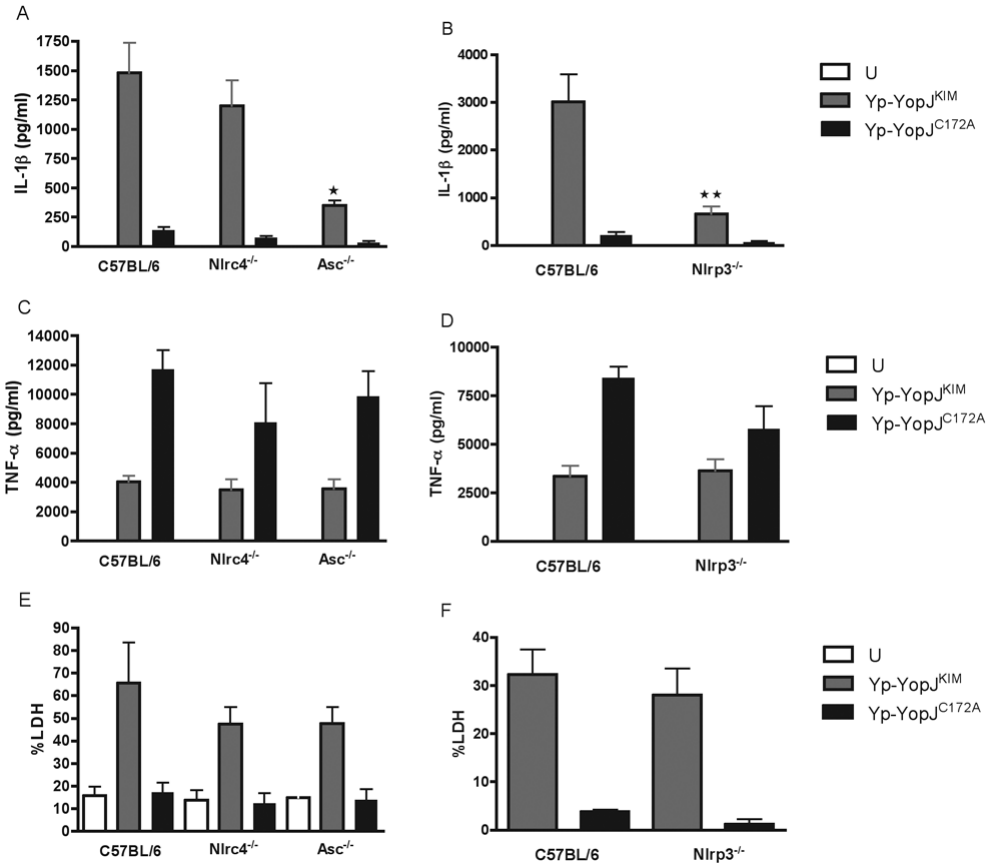
Determination of the importance of inflammasome components for cytokine secretion and cell death in infected macrophages. Wild-type BMDMs, or BMDMs deficient for NLRC4 (Ipaf), ASC (ASC) or NLRP3 (Nalp3) were left uninfected (U) or infected with Yp-YopJ^KIM^ or Yp-YopJ^C172A^ at an MOI of 10. Supernatants were collected at 24 hr post-infection and analyzed by ELISA to quantify amounts of secreted IL-1β (A, B) or TNF-α (C, D). Cell death was measured by LDH release (E, F). Results shown are the average of three independent experiments. Error bars represent standard deviation. Statistical significance compared to YP-YopJ^KIM^-infected wild-type BMDMs was determined (ANOVA; P<0.05, *; P<0.01, **).

The importance of several different inflammasome components for *Y. pestis*-induced secretion of IL-1β and IL-18 was investigated using NLRP3 (Nlrp3^-/-^)-, ASC (Asc^-/-^)- or NLRC4 (Nlrc4^-/-^)-deficient BMDMs. The mutant BMDMs or wild-type control macrophages were infected with Yp-YopJ^KIM^ or Yp-YopJ^C172A^. Tissue culture supernatants were collected and analyzed by ELISA to measure the levels of IL-1β and IL-18 present after 24 hr of infection. NLRP3- or ASC-deficient BMDMs infected with Yp-YopJ^KIM^ secreted significantly lower levels of IL-1β and IL-18 as compared to wild-type macrophages infected with Yp-YopJ^KIM^ (Figure 7A,B; Figure S7A, B in Text S1). NLRC4-deficient macrophages released similar levels of these cytokines as compared to wild-type BMDMs (Figure 7A, Figure S7A in Text S1), suggesting that NLRC4 does not play a significant role in caspase-1 activation and cytokine secretion during Yp-YopJ^KIM^ infection. Both Yp-YopJ^KIM^ and Yp-YopJ^C172A^ stimulated infected BMDMs to secrete TNF-α, although higher levels (∼2 to 3 fold) of TNF-α were secreted from macrophages infected with Yp-YopJ^C172A^ regardless of macrophage type infected (Figure 7C, D). Thus, NLRP3 and ASC, but not NLRC4, are involved in the secretion of IL-1β and IL-18, but not TNF-α, from Yp-YopJ^KIM^ -infected macrophages.

### YopJ^KIM^-induced macrophage apoptosis does not require NLRP3, ASC or NLRC4

To determine if NLRP3, NLRC4 or ASC play a role in YopJ^KIM^-dependent apoptosis, wild-type BMDMs or BMDMs deficient for these inflammasome components were left uninfected or infected with Yp-YopJ^KIM^ or Yp-YopJ^C172A^. Tissue culture supernatants were collected 24 hr post-infection and analyzed for LDH. Similar levels of LDH were released from NLRP3, NLRC4 or ASC-deficient BMDMs as compared wild-type macrophages after Yp-YopJ^KIM^ infection (Figure 7E, F). Low levels of LDH release occurred in all macrophages infected with Yp-YopJ^C172A^. These results demonstrate that apoptosis can occur in Yp-YopJ^KIM^ -infected macrophages in the absence of NLRP3, NLRC4 or ASC, consistent with our previous data showing that macrophage apoptosis during Yp-YopJ^KIM^ infection is independent of caspase-1 [8].

### Evidence that K^+^ efflux is important for secretion of IL-1β and IL-18 from macrophages infected with Yp-YopJ^KIM^

**Figure 8:**
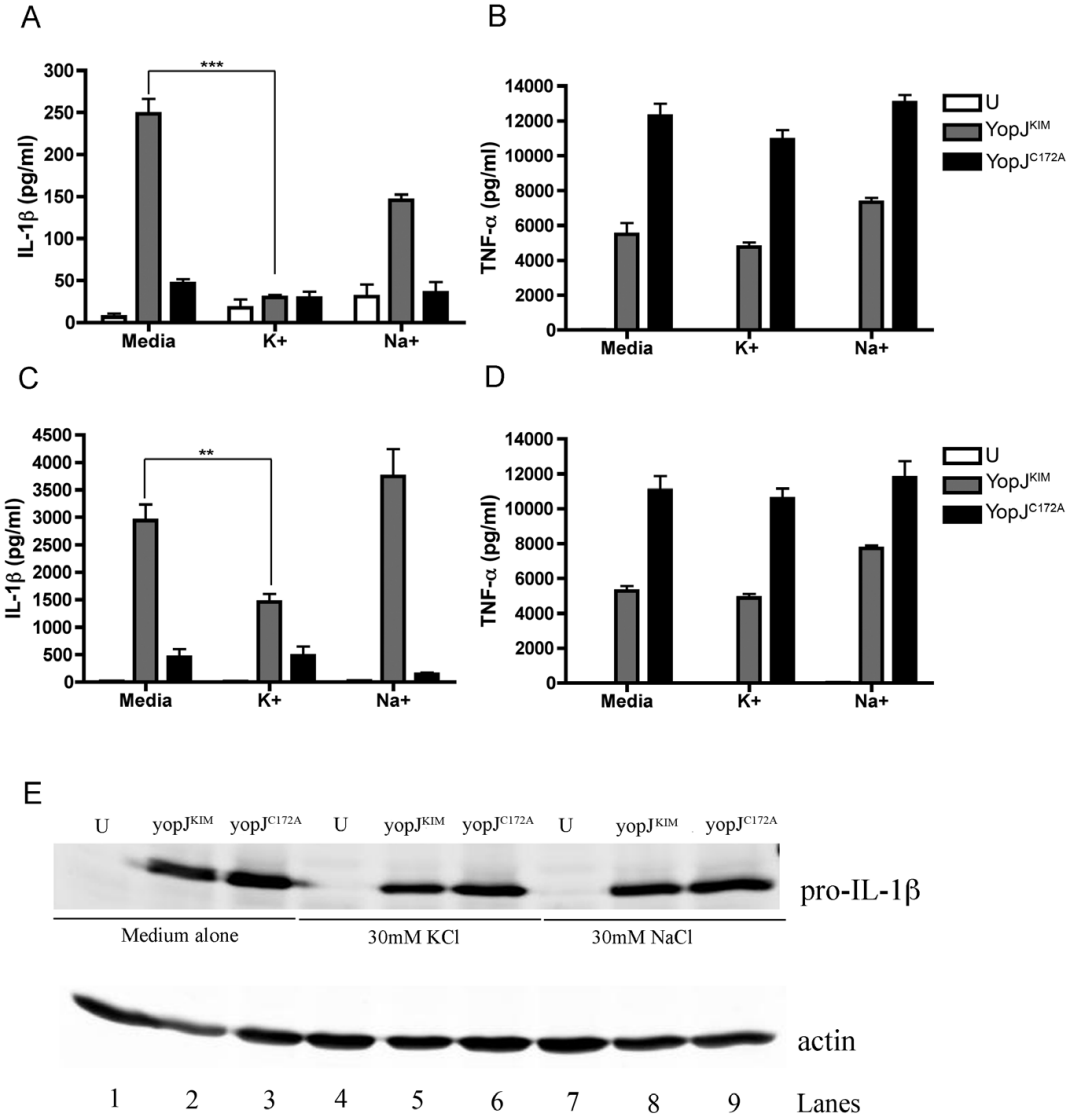
Determination of the effect of exogenous KCl on cytokine expression and secretion in infected macrophages. BMDMs were left uninfected (U) or infected at an MOI of 10 with Yp-YopJ^KIM^ or Yp-YopJ^C172A^ and treated with 30 mM KCl (K+), 30 mM NaCl (Na+) or left untreated (Media) as indicated. Supernatants were collected at 8 hr (A, B) or 24 hr (C, D) post-infection, and secreted IL-1β (A, C) and TNF-α (B, D) were measured by ELISA. (E) Pro-IL-1β in cell lysates prepared at 8 hr post-infection was detected by immunoblotting with anti-IL-1β antibody. Actin in the same samples was detected by immunoblotting as a loading control. Results shown in A–D are the average of three independent experiments. Error bars represent standard deviation. Statistical significance compared to YP-YopJ^KIM^-infected BMDMs in media alone was determined (ANOVA; P<0.01, **; P<0.001, ***).

Efflux of intracellular K^+^ has been implicated in the activation of the NLRP3/ASC/caspase-1 inflammasome [10], [11], [12]. To assess a role for intracellular K^+^ efflux in caspase-1 activation and IL-1β release during infection with *Y. pestis*, BMDMs were infected with Yp-YopJ^KIM^ or Yp-YopJ^C172A^, and then incubated in cell culture media supplemented with 30 mM KCl, 30 mM NaCl or no supplement. Cell culture supernatants were collected at 8 hr and 24 hr time points and analyzed for the presence of IL-1β and TNF-α by ELISA. Significantly lower levels of IL-1β (∼5-fold) were secreted from macrophages infected with Yp-YopJ^KIM^ in the presence of 30 mM KCl as compared to untreated macrophages at 8 hr post-infection (Figure 8A). Macrophages infected with Yp-YopJ^KIM^ in the presence of 30 mM NaCl appeared to secrete IL-1β to slightly lower levels as compared to untreated infected macrophages at 8 hr post-infection, but this difference was not significant (Figure 8A). A similar trend of IL-1β secretion was observed at the 24 hr time point when macrophages were infected with Yp-YopJ^KIM^ in the presence or absence of KCl or NaCl (Figure 8C). Macrophages infected with Yp-YopJ^C172A^ secreted similar low levels of IL-1β regardless of treatment (Figure 8A, C). Secretion of TNF-α from Yp-YopJ^KIM^- or Yp-YopJ^C172A^-infected macrophages was not affected by the presence of 30 mM KCl or NaCl (Figure 8B, D). In addition, the presence of 30 mM KCl did not diminish LDH release from BMDMs infected with Yp-YopJ^KIM^ (data not shown). BMDMs deficient for the purinergic receptor, P2X_7_, secreted similar levels of IL-1β and IL-18 as did wild-type macrophages infected with Yp-YopJ^KIM^, indicating that this receptor does not play a significant role in inducing the secretion of these cytokines (data not shown). Taken together, these results suggest that a K^+^ efflux that occurs independent of P2X_7_R is important for activation of the NLRP3/ASC/caspase-1 inflammasome in macrophages infected with Yp-YopJ^KIM^.

To examine how *Y. pestis* infection and KCl treatment affected steady state levels of pro-IL-1β, lysates of macrophages left untreated or treated with KCl or NaCl were prepared at 8 hr post-infection and analyzed by immunoblotting for pro-IL-1β or actin as a loading control. As shown in Figure 8E, infection stimulated production of pro-IL-1β, with steady state levels of pro-IL-1β slightly lower in macrophages infected with Yp-YopJ^KIM^ as compared to Yp-YopJ^C172A^ (compare lanes 2 and 3, 5 and 6 and 8 and 9). Similar amounts of pro-IL-1β were detected in macrophages infected with Yp-YopJ^KIM^ in the absence or presence of 30 mM KCl or 30 mM NaCl (Figure 8E, compare lane 2 with 5 and 8). These results indicated that reduced detection of IL-1β in supernatants of macrophages infected with YopJ^KIM^ and treated with exogenous KCl was not due to KCl inhibiting production of pro-IL-1β.

## Discussion

It was previously shown that caspase-1 was activated during YopJ-induced apoptosis of macrophages infected with *Y. pseudotuberculosis* [6]. In addition, it was demonstrated that YopJ^KIM^ had increased capacity to cause macrophage apoptosis and activate caspase-1 as compared to other YopJ isoforms [8]. However, the mechanism of YopJ-induced caspase-1 activation and the molecular basis for enhanced apoptosis and activation of caspase-1 in macrophages by YopJ^KIM^ was unknown. The results of studies reported here indicate that several of the requirements for YopJ-induced apoptosis and caspase-1 activation are the same, and therefore it is likely that these two processes are mechanistically connected. First, it is known that TLR4 signaling is important for YopJ-induced macrophage apoptosis [21], [22], [23], [24] and we show here that the two major TLR adaptors, MyD88 and Trif, are important for YopJ-induced caspase-1 activation. Second, desensitization of macrophages by pretreatment with LPS decreases YopJ-induced apoptosis [38] and caspase-1 activation. Third, comparison of the activities of different YopJ isoforms showed a direct correlation between apoptosis, caspase-1 activation and inhibition of MAPK and NF-κB signaling pathways. Forth, when macrophages in which *Ikk*β was conditionally deleted were infected with *Y. pestis*, caspase-1 activation increased, providing genetic evidence that IKKβ is an important target of YopJ for caspase-1 activation, as well as apoptosis [25].

Inhibition of MAPK and NF-κB pathways by YopJ is thought to reduce expression of survival factors (e.g. FLIP, XIAP), thereby potentiating TLR4 signaling to trigger apoptosis [22], [23], [24]. Inactivation of the MAPK and NF-κB pathways by YopJ could also prevent expression of putative negative regulators of caspase-1 (e.g. PAI-2) [7]. It is important to point out that there is no direct evidence that PAI-2 inhibits caspase-1 activation independently of blocking apoptosis, rather the data show that PAI-2 overexpression reduces both apoptosis and caspase-1 activation [7]. It is possible that PAI-2 inhibits apoptosis and that events triggered downstream of TLR4-dependent programmed cell death are required for caspase-1 activation. We suggest that caspase-1 activation is a normal outcome of a type of apoptosis that is triggered in naïve macrophages by TLR4 signaling combined with pathogen interference with MAPK and NF-κB pathways.

Data presented here suggest that YopJ^KIM^ triggers increased apoptosis and caspase-1 activation because it is a better inhibitor of macrophage survival pathways than other YopJ isoforms. YopJ^KIM^ could function as a better inhibitor of macrophage signaling pathways if it had a longer half-life in the host cell, or had higher affinity for substrates. The F177L polymorphism could increase protein stability, although it is not immediately clear why a Leu at position 177 rather than a Phe would increase protein half-life. The K206E mutation could increase half-life, which is reasonable since Lys residues can be subject to ubiquitination. Although not mutually exclusive of the preceding ideas, we favor the hypothesis that the F177L and K206E substitutions allow YopJ^KIM^ to bind more tightly to substrates, thereby making acetylation of targets more efficient at limiting enzyme concentrations. We obtained two pieces of evidence supporting this hypothesis. First, YopJ^KIM^ had higher apparent affinity for IKKβ than YopJ^CO92^ when these interactions were measured in cell lysates by a GST pull down assay. Second, macrophages infected with Yp-YopJ^KIM^ had lower levels of phosphorylated IκBα and MAPKs as compared to macrophages infected with Yp-YopJ^CO92^, indicating that there was increased inhibition of IKKβ and MAPK kinase activity by Yp-YopJ^KIM^.

The results suggest a model whereby the canonical *yopJ* allele in *Y. pseudotuberculosis* (*yopJ^YPTB^*) was inherited by an ancestral *Y. pestis* strain, from which it evolved to encode an isoform with higher apoptotic and caspase-1-activating potential, YopJ^KIM^, by the F177L mutation. The predicted sequence of a YopJ protein in *Y. pestis* biovar 2.MED strain K1973002 (ZP_02318615) is identical to the sequence of YopJ^KIM^, suggesting that the phenotype observed is not an artifact resulting from a mutation acquired during laboratory passage, but is associated with a unique *yopJ* genotype associated with 2.MED strains. It is also hypothesize that the *yopJ^CO92^* allele evolved from *yopJ^YPTB^* to encode an isoform with lower cytotoxic and caspase-1 activating potential (YopJ^CO92^) by the E206K codon substitution. How these polymorphisms in YopJ affect *Y. pestis* virulence and or the host response is not known but is an important question to address in future studies.

The importance of different inflammasome components for YopJ-dependent activation of caspase-1 in macrophages infected with *Y. pseudotuberculosis* has recently been examined [6]. This study showed that NLRP3 and ASC were not required for activation of caspase-1 as measured by immunoblot analysis of caspase-1 cleavage [6]. Those results would appear to be in conflict with findings presented here showing a role for NLRP3 and ASC in secretion of IL-1β and IL-18 from macrophages infected with Yp-YopJ^KIM^. However, recent studies suggest that multiple distinct caspase-1 activation pathways with different biological outcomes can operate in macrophages infected with a bacterial pathogen. For example, evidence has been obtained that *Legionella pneumophila* stimulates two distinct pathways of caspase-1 activation in macrophages [46]. ASC is required for secretion of active IL-18 from *L. pneumophila*-infected macrophages, but is not required for caspase-1 dependent induction of pyroptosis [46]. In addition, the multiplicity and temporal stage of infection of macrophages with a bacterial pathogen can affect the requirements for cell death and activation of caspase-1. *Shigella flexneri* infection of macrophages at low MOI (<10) for short periods of time induces NLRC4-dependent pyroptosis [47], [48], while infection at higher MOI (50) for longer time periods induces NLRP3-dependent pyronecrosis [48]. Two different infection procedures for examining YopJ-induced caspase-1 activation in macrophages have been used in this study and previous publications [6], [8]. A high MOI (20) followed by 1 hr of bacterial-host cell contact before addition of gentamicin results in detectable YopJ-dependent apoptosis and caspase-1 activation within 2 hr of infection (Figure 5A, Figure S3 in Text S1) [6] but no detectable secretion of IL-1β by this time point (data not shown) [6] . A low MOI (10) followed by 20 min of bacterial-host cell contact before addition of gentamicin results in detectable apoptosis and caspase-1 activation by 8–9 hr (Figure 5B,C) [8], at which time secreted IL-1β and IL-18 are first detected [8]. High amounts of secreted IL-1β and IL-18 are detected at 24 hr post infection under the low MOI procedure (e.g. Figure 2) [8]. The high and low MOI infection procedures may result in different requirements for NLRs to activate caspase-1, as shown by a requirement for ASC and NLRP3 in the latter but not former method. Interestingly, the low MOI procedure appears to slow down the kinetics of apoptosis and caspase-1 activation, which is likely important to allow for synthesis of NLRP3 [19] and the pro-forms of IL-1β and IL-18.

Under the low MOI conditions the presence of 30 mM KCl in the infection medium inhibited the secretion of IL-1β and IL-18 from macrophages infected with Yp-YopJ^KIM^, suggesting an important role for K^+^ efflux in caspase-1 activation. Efflux of intracellular K^+^ mediated by the P2X_7_R is critical for ATP-induced caspase-1 activation in macrophages primed with LPS [49]. However, like other NLRP3 activators such as nigericin, caspase-1 activation in response to Yp-YopJ^KIM^ infection did not require P2X_7_R. One possibility is that pore formation during YopJ^KIM^-induced apoptosis leads to K^+^ efflux, resulting in activation of the NALP3/ASC/caspase-1 inflammasome. One limitation of this model is that it remains to be determined if K^+^ efflux acts as a proximal activating signal of the NALP3/ASC/caspase-1 inflammasome. A second limitation of this model is that apoptosis is generally associated with maintenance of an intact plasma membrane, until late stages of cell death [4]. Future experiments will need to address the possibility that YopJ-induced apoptosis of *Yersinia*-infected macrophages can be associated with rapid membrane permeability, resulting in K^+^ efflux and caspase-1 activation.

## Materials and Methods

### Ethics statement

All animal use procedures were conducted following the NIG Guide for the Care and Use of Laboratory Animals and performed in accordance with Institutional regulations after review and approval by the Institutional Animal Care and Use Committee at Stony Brook University.

### *Yersinia* strains, plasmids and growth conditions

*Y. pestis* and *Y. pseudotuberculosis* strains used in this study are listed in Table S1 in Text S1. *Y. pestis* strains used in this study are derived from KIM5 [8], which lacks the pigmentation locus (*pgm*) and are exempt from select agent guidelines and conditionally attenuated. Introduction of codon changes into *yopJ* in KIM5 (Table S1 in Text S1) was performed using the suicide plasmid pSB890 and allelic exchange as described [50]. The arabinose inducible plasmid encoding YopJ^KIM^ (pYopJ-GSK) has been described [43]). Codon changes were introduced into *yopJ^KIM^* on this plasmid using Quikchange (Invitrogen), yielding pYopJ^YPTB^-GSK, pYopJ^KIME206K^-GSK, and pYopJ^CO92^-GSK. The resulting plasmids were used to transform IP26 (IP2666 Δ*yopJ*) using electroporation and selection on LB agar plates containing ampicillin (100 µg/ml) [8].

### Bone marrow macrophage isolation and culture conditions

Bone marrow derived macrophages (BMDM) were isolated from the femurs of 6- to 8-week-old C57BL/6 female mice (Jackson Laboratories), *Casp-1^-/-^* mice [8], P2X_7_ receptor-deficient mice [51], *Ikkβ^f/f^* or *Ikkβ^f/f^;MLysCre* mice [52], [53], NLRC4- (Nlrc4^-/-^), ASC- (Asc^-/-^) or NLRP3- (Nlrp3^-/-^) deficient mice [54], and MyD88-, Trif- and MyD88/Trif-deficient mice [55] and cultured as previously described [56], [57].

### Macrophage infections for LDH release, cytokine ELISA, IL-1β immunoblotting and FLICA

*Y. pestis* cultures were grown overnight with aeration in HI broth at 28°C. The next day the cultures were diluted to an OD_600_ of 0.1 in the same medium supplemented with 2.5 mM CaCl_2_ and incubated for 2 hr at 37°C with aeration. Twenty-four hours before infection, BMDM were seeded into wells of 24-well plates at a density of 1.5×10^5^ cells/ml. Macrophage infections were performed in 37°C incubators with 5% CO_2_ at a multiplicity of infection (MOI) of 10 as previously described [8]. After addition of bacteria, plates were centrifuged for 5 minutes at 95 xg to induce contact between bacteria and macrophages. After incubation at 37°C for 15 minutes, macrophages were washed once with pre-warmed PBS to remove any bacteria that have not been taken up. Fresh infection medium containing 8 µg/ml of gentamicin was added for 1 hr at 37°C. After 1 hr, macrophages were washed once with PBS and a lower concentration of gentamicin (4.5 µg/ml) in fresh tissue culture media was added for the remaining incubation times. To inhibit potassium efflux from infected macrophages, potassium chloride (KCl) was added to a final concentration of 30 mM concurrently with the media exchanges containing 8 µg/ml gentamicin and 4.5 µg/ml gentamicin [8]. Sodium chloride (NaCl) was used as a control and added as above at a concentration of 30 mM above baseline. Amounts of IL-1β, TNF-α or IL-18 secreted into tissue culture media during infection assays were measured by ELISA as described [8]. Supernatants from infected macrophages were collected and analyzed for LDH release as described [8]. Staining with 6-carboxyfluorescein–YVAD–fluoromethylketone (FAM-YVAD-FMK; fluorescent inhibitor of apoptosis (FLICA)) (Immunochemistry Technologies) to detect active caspase-1 in infected macrophages was performed using fluorescence and phase microscopy as described [8] with the exception that the procedure was performed 9 hr post-infection, and the anti-*Yersinia* immunolabeling step was omitted. Quantification of percent caspase-1 positive BMDMs was performed by scoring macrophages for positive signal in three different randomly selected fields (∼50–100 cells per field) on a coverslip.

### Immunoblotting for pro-IL-1β

At 8 hr post-infection, macrophage lysates from triplicate wells were collected in 100 µl of 1X lysis buffer (50 mM Tris-HCl, 5 mM EDTA, 150 mM NaCl, 1% Triton X-100, 2 mM DTT and a protease inhibitor cocktail [Complete Mini, EDTA-Free, Roche]). Proteins were resolved by SDS-PAGE and transferred to a nitrocellulose membrane. To detect IL-1β, membranes were blotted with goat anti-IL-1β (R&D Systems). A secondary antibody, Hamster anti-goat IRDye 700 antibody (Rockland) was used to detect samples, and blots were viewed on the Odyssey Infrared Imaging System (LI-COR). To control for loading, blots were probed with a rabbit anti-actin antibody (Sigma-Aldrich).

### Phospho-IκBα ELISA

BMDMs (10^6^ cells per well) were seeded in 6-well plates. *Y. pestis* cultures were grown as above and used to infect BMDM at a MOI of 50. 1 hr post infection, cells were washed with ice-cold PBS and incubated in 150 ul of 1X Lysis Buffer (Cell Signaling) for 5 min. Cells were scraped on ice and sonicated twice for 5 seconds each. Lysates were centrifuged at 4°C for 10 min and 100 µl of supernatant was used for ELISA. Phospho-IκBα levels were determined using a PathScan Phospho-IkappaB-alpha (Ser32) Sandwich ELISA kit according to manufacturer's protocol (Cell Signaling).

### Macrophage infections for Phospho-MAPK ELISA

BMDMs (10^6^ cells per well) were seeded in 6-well plates. *Y. pestis* cultures were grown in HI at 28°C overnight and diluted 1∶20 next day in the same medium supplemented with 20 mM NaOX and 20 mM MgCl2. Cultures were shaken at 28°C for 1 hr and switched to 37°C for 2 hr. Cells were infected at an MOI of 20 and incubated for 30 or 60 min without adding gentamicin. Macrophages were harvested and lysed as above. The PathScan MAP Kinase Multi-Target Sandwich ELISA kit was used to determine phosphor-ERK, -p38 and –JNK levels according to manufacturer's instruction (Cell Signaling).

### Macrophage infections for YopJ translocation and caspase-1 cleavage assays

*Y. pseudotuberculosis* strains were grown in 2xYT at 26°C overnight and diluted 1∶40 in the same medium supplemented with 20 mM NaOX, and 20 mM MgCl_2_. Cultures were shaken at 26°C for 1 hr and shifted to 37°C for 2 hr. BMDMs were seeded into wells of 6-well plates at a density of 10^6^ cells/well. Bacteria were harvested, washed with DMEM and added to BMDMs at an MOI of 20. After 1 hr of infection gentamicin was added to a final concentration of 100 µg/ml. To induce expression of YopJ-GSK proteins, arabinose (0.2%) was maintained during grown in 2xYT at 37°C and in the cell culture medium used for infection. *Y. pestis* strains were grown and used to infect macrophages as above except that HI broth was used and arabinose was omitted. Two hr post-infection, infected BMDMs were washed with PBS and lysed in buffer containing 50 mM Tris-HCl pH 8.0, 5 mM EDTA, 2% Triton X-100, and 0.02% sodium azide with protease inhibitors. In some experiments the macrophages were incubated with 50 ng/ml of LPS for 3 hrs and then exposed to ATP at final concentration of 2.5 mM for 1 hr as a positive control for caspase-1 cleavage. Proteins were resolved by 10% SDS-PAGE, transferred to a PVDF membrane and probed with anti-phospho-GSK-3β primary antibody (Cell Signaling). In some experiments the blots were stripped and re-probed with rabbit polyclonal anti-caspase-1 antibodies (Santa Cruz) or directly developed with this antibody. As a loading control blots were reprobed with an anti-actin antibody (Sigma-Aldrich, clone AC15). Goat anti-rabbit HRP conjugated secondary antibody was used. Blots were detected with ECL reagent (Perkin Elmer Life Sciences, Inc.).

### GST pull down assay of YopJ^KIM^-IKKβ interaction

Plasmids for expression of GST-YopJ fusion proteins were constructed from pLP16 [58]. The pLP16 vector was derived from pGEX-2T and codes for YopJ^YPTB^ with an N-terminal glutathione-S transferase (GST) affinity tag and a C-terminal M45 epitope tag. Quikchange mutagenesis (Invitrogen) was used to introduce codon changes into pLP16 to generate pGEX-2T-YopJ^KIM^, pGEX-2T-YopJ^KIMC172A^ and pGEX-2T-YopJ^CO92^, which encode GST-YopJ^KIM^, GST-YopJ^C172A^ and GST-YopJ^CO92^, respectively. The plasmids pGEX-2T, pGEX-2T-YopJ^KIM^, pGEX-2T-YopJ^KIMC172A^ and pGEX-2T-YopJ^CO92^ were used to transform *E. coli* TUNER cells (Novagen). Cultures of TUNER cells harboring the above plasmids were grown in LB at 37°C to OD600 of 0.2. IPTG was added to 0.1 mM final concentration and cultures were grown at 18°C with shaking for 4 hrs. The bacterial pellet obtained from 40 ml of each culture was resuspended in PBS supplemented with protease inhibitor cocktail (Roche) and sonicated on ice. The solubility of proteins in the sonicates was increased by incubation in the presence of a buffer containing 10% sarkosyl at 4°C overnight [59]. After centrifugation, the supernatant of the bacterial lysate was diluted 5 times with a buffer containing 4% Triton X-100 and 40 mM CHAPS at final concentrations. Thirty µl of glutathione beads (GST Bind Kit, Novagen) were added and the mixture was shaken at 4°C for 1 hr. Beads were washed 4 times with 1 ml of GST Bind Kit buffer and used for pull down assays.

Cell lysates containing overexpressed IKKβ were prepared from HEK293T cells tranfected with a retroviral construct (pCLXSN-IKKβ-IRES-GFP) [25]. HEK293T cells were seeded in 10 cm dishes and grown to reach 70% confluence. The culture medium was replaced with serum free DMEM and the HEK293T cells in each dish were transfected with 10 µg of pCLXSN-IKKβ-IRES-GFP using a calcium phosphate method. Six hrs post transfection, the culture medium was replaced with DMEM containing 10% FBS. Cells were harvested 48 hrs post transfection, sonicated in PBS and centrifuged. Supernatants were stored at −80°C until use.

Beads containing bound GST proteins were incubated with 250 µl of cell lysate supernatants from transfected HEK293T supernatant for 4 hrs at 4°C with constant rotation. The beads were then washed 4 times with 1 ml of PBS each and proteins bound to the beads were eluted in boiling 2X Laemmli sample buffer. Samples of the eluates were subjected to SDS-PAGE and immunoblotting. Rabbit polyclonal anti-IKKβ antibodies and mouse monoclonal anti-GST antibodies were purchased from Cell Signaling and Santa Cruz, respectively. Immunoblot signals representing IKKβ and GST or GST fusion proteins were quantified using an Odyssey imaging system.

### Statistical analysis

Experimental data analyzed for significance (GraphPad Prism 4.0) were performed three independent times. Probability (P) values for multiple comparisons of cytokine, phospho-IκBα ELISA and LDH release data were calculated by one-way ANOVA and Tukey's multiple comparisons post-test. P values for two group comparisons of cytokine, phospho-IκBα, and phospho-MAPK ELISA were calculated by two-tailed paired student t test. P values were considered significant if less than 0.05.

## Supporting Information

Text S1 The supporting text includes the supplemental Table S1, Figures S1-S7, and supplemental experimental procedures.
(DOC)
