# Supplementary material for: A Yersinia Effector with Enhanced Inhibitory Activity on the NF-κB Pathway Activates the NLRP3/ASC/Caspase-1 Inflammasome in Macrophages
Source: PLoS Pathog. 2011 Apr 21;7(4):e1002026. doi: 10.1371/journal.ppat.1002026 (PMC3080847; doi:10.1371/journal.ppat.1002026)
Supplement: Text S1 — The supporting text includes the supplemental Table S1, Figures S1-S7, and supplemental experimental procedures. (DOC) [file ppat.1002026.s001.doc]

**Supporting text**

**A *Yersinia* effector with enhanced inhibitory activity on the NF-κB pathway activates the NLRP3/ASC/caspase-1 inflammasome in macrophages**

Ying Zheng, Sarit Lilo, Igor E. Brodsky, Yue Zhang, Ruslan Medzhitov, Kenneth B. Marcu, and James B. Bliska

**Table S1. *Yersinia*** strains used in this study

| **Strain name** | **Relevant Characteristics** | **Reference or source** |
| --- | --- | --- |
| ***Y. pestis*** |  |  |
| Yp-YopJ^KIM^ | KIM5, Biovar 2.MED, (pCD1Ap, pMT1^+^, pPCP1^+^, Δ*pgm,* Ap^r^) | [1] |
| Yp-YopJ^C172A^ | KIM5 pCD1Ap *yopJC172A* (codon change of Cys172 to Ala172), Ap^r^ | [1] |
| Yp-YopJ^CO92^ | KIM5 pCD1Ap *yopJL177F E206K* (codon change of Leu177 to Phe177, Glu206 to Lys206), Ap^r^ | This Study |
| Yp-YopJ^YPTB^ | KIM5 pCD1Ap *yopJL177F* (codon change of Leu177 to Phe177), Ap^r^ | This Study |
| Yp-YopJ^KIME206K^ | KIM5 pCD1Ap *yopJE206K* (codon change of Glu206 to Lys206), Ap^r^ | This Study |
| ***Y. pseudotuberculosis*** |  |  |
| IP2666 | Serogroup O3 (pYV^+^) |  |
| IP26 | IP2666 pYV *yopJΔ1-867* | [1] |


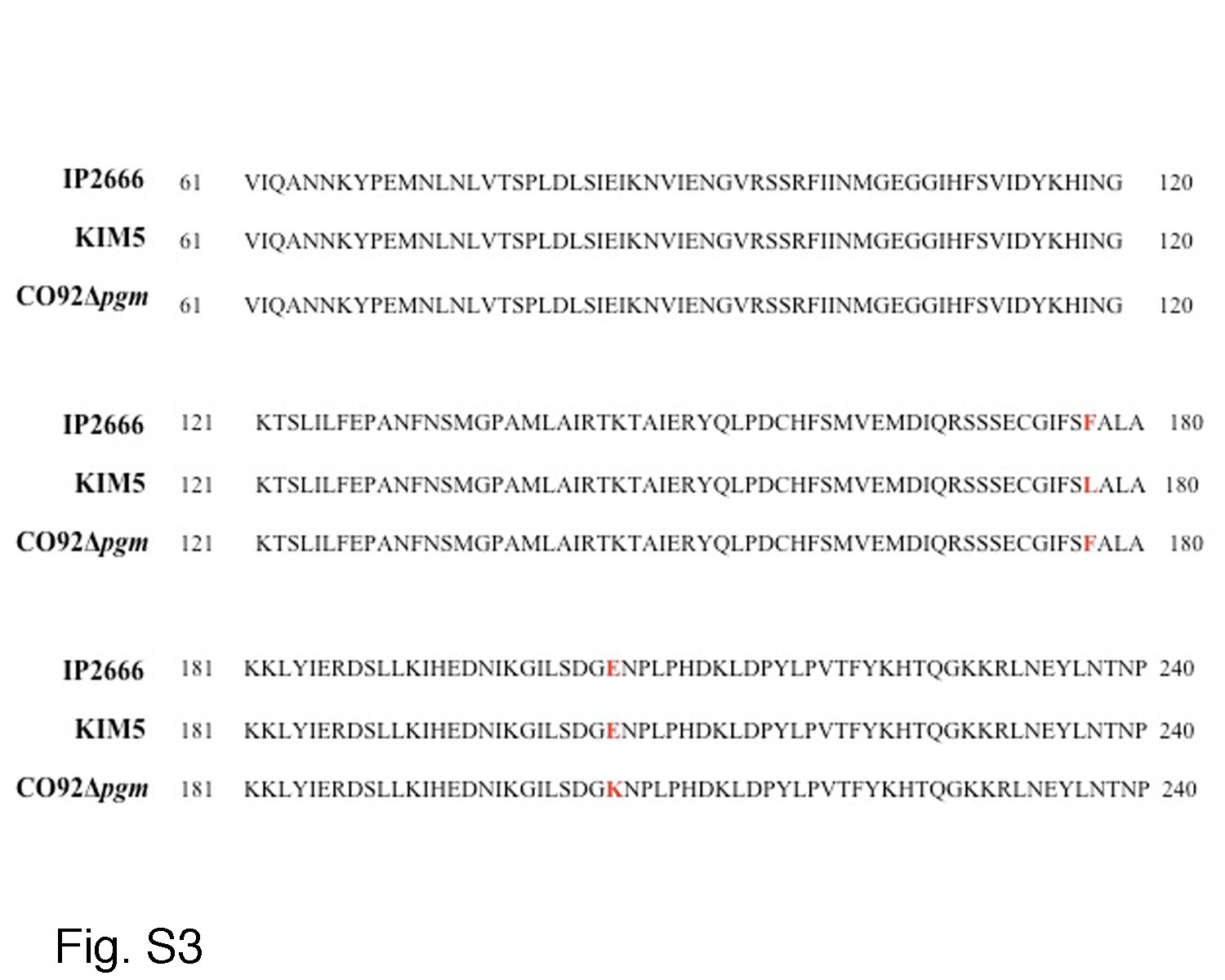


**Figure S1. Alignment of the amino acid sequence of YopJ from different *Yersinia* strains.** Predicted protein sequence alignment of YopJ proteins from *Y. pseudotuberculosis* strain IP2666 and *Y. pestis* strains KIM5 and CO92*Δpgm*. Shown are amino acids 61-240 out of 288 total. Red letters display amino acid differences at positions 177 and 206. The predicted catalytic core corresponds to residues 109 to 194.


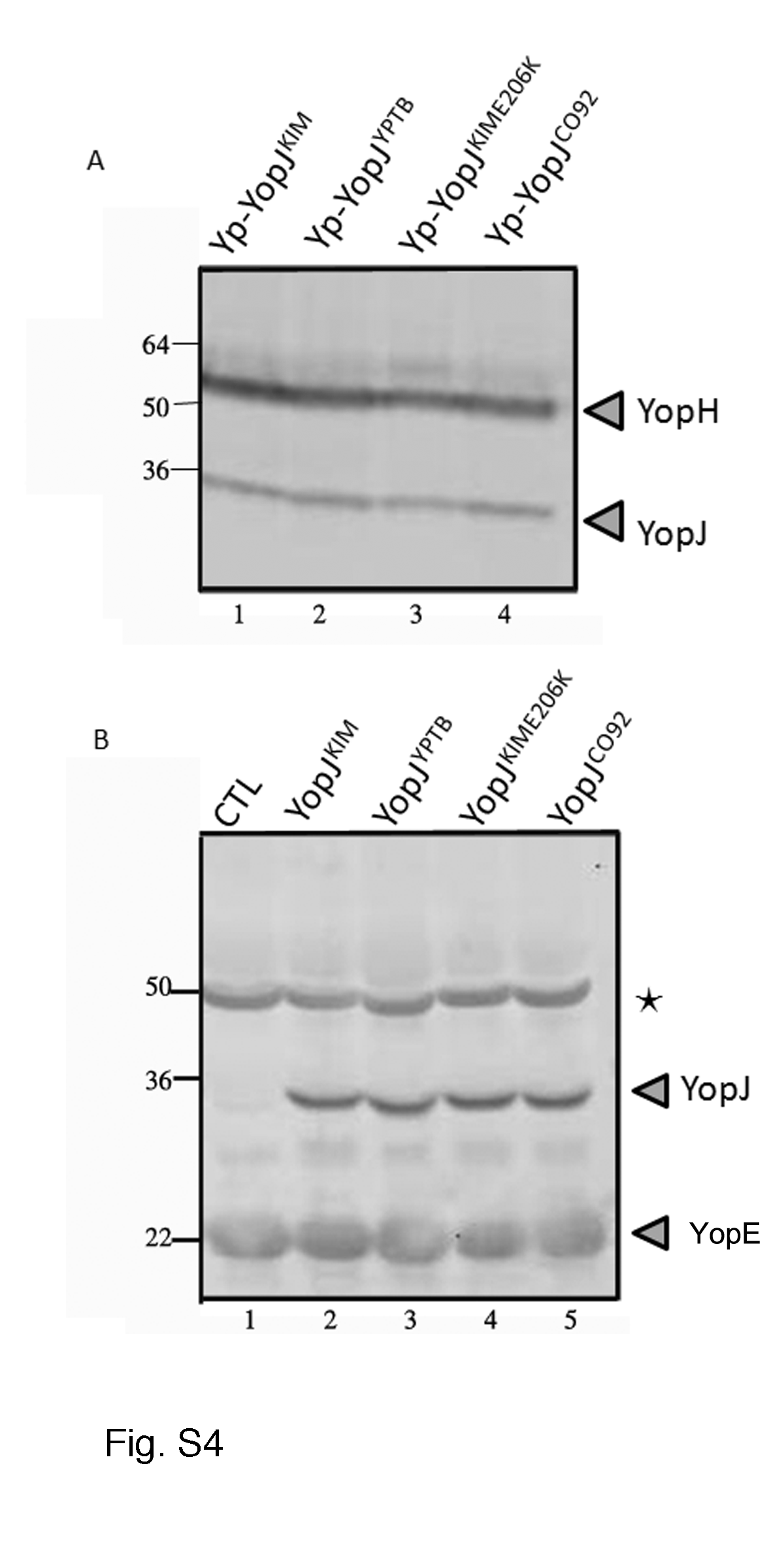


**Figure S2. Different YopJ isoforms show equal secretion profiles.** *Y. pseudotuberculosis* IP26 (IP2666*ΔyopJ* ) strains harboring pBAD plasmids expressing GSK-tagged YopJ^KIM^ (lanes 1 and 2), YopJ^YPTB^ (lane 3), YopJ^KIME206K^ (lane 4), or YopJ^CO92^ (lane 5), were induced to secrete Yops in low Ca^2+^ LB broth in the absence (lane 1, CTL) or presence (lanes 2-5) of arabinose. Bacterial broth supernatants were precipitated with TCA and dissolved in 1× Laemmli buffer. Samples were processed for SDS-PAGE and immunoblotting. Immunoblots were probed using anti-GSK and anti-YopE antibodies. Asterisk denotes non-specific signal. Positions of YopJ and YopE are shown on the right. Positions of molecular weight standards (kDa) are shown on the left.

**
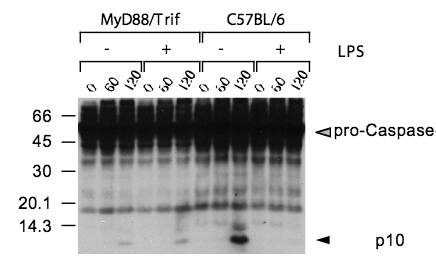
**

**Figure S3. MyD88 or Trif are required for caspase-1 activation in macrophages infected with *Yersinia.*** BMDMs from C57BL/6 mice deficient for MyD88 and Trif or from wild-type C57BL/6 mice were left untreated or treated with LPS for 3 hr. The BMDM were then left uninfected (time 0) or infected with wild-type *Y. pseudotuberculosis* IP2666 for 60 or 120 min at MOI of 20. Lysates of infected BMDMs were prepared and analyzed by immunoblotting with anti-caspase-1 antibody. Positions of pro-caspase-1 and cleaved caspase-1 (p10) are shown on the right. Positions of molecular weight standards (kDa) are indicated on the left.

**
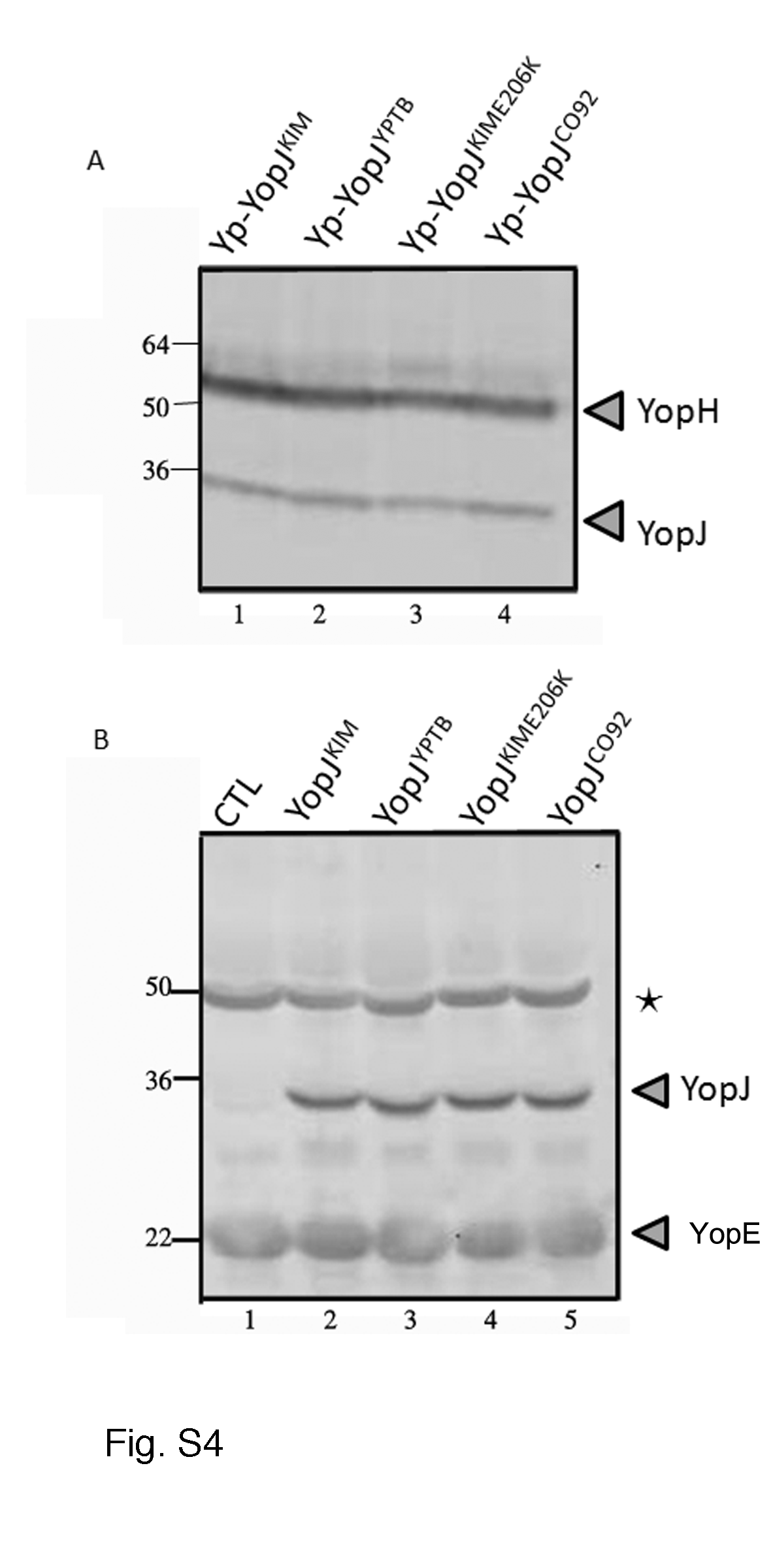
**

**Figure S4. Different YopJ isoforms show equal expression profiles.** The indicated Yp-YopJ strains were grown at 37°C in TMH media containing 2.5mM Ca^2+^, normalized by OD_600_ and lysed in 1× Laemmli buffer. Samples were analyzed by immunoblotting using monoclonal anti-YopJ and anti-YopH monoclonal antibodies. Positions of YopH and YopJ are shown on the right. Positions of molecular weight standards (kDa) are shown on the left.

**
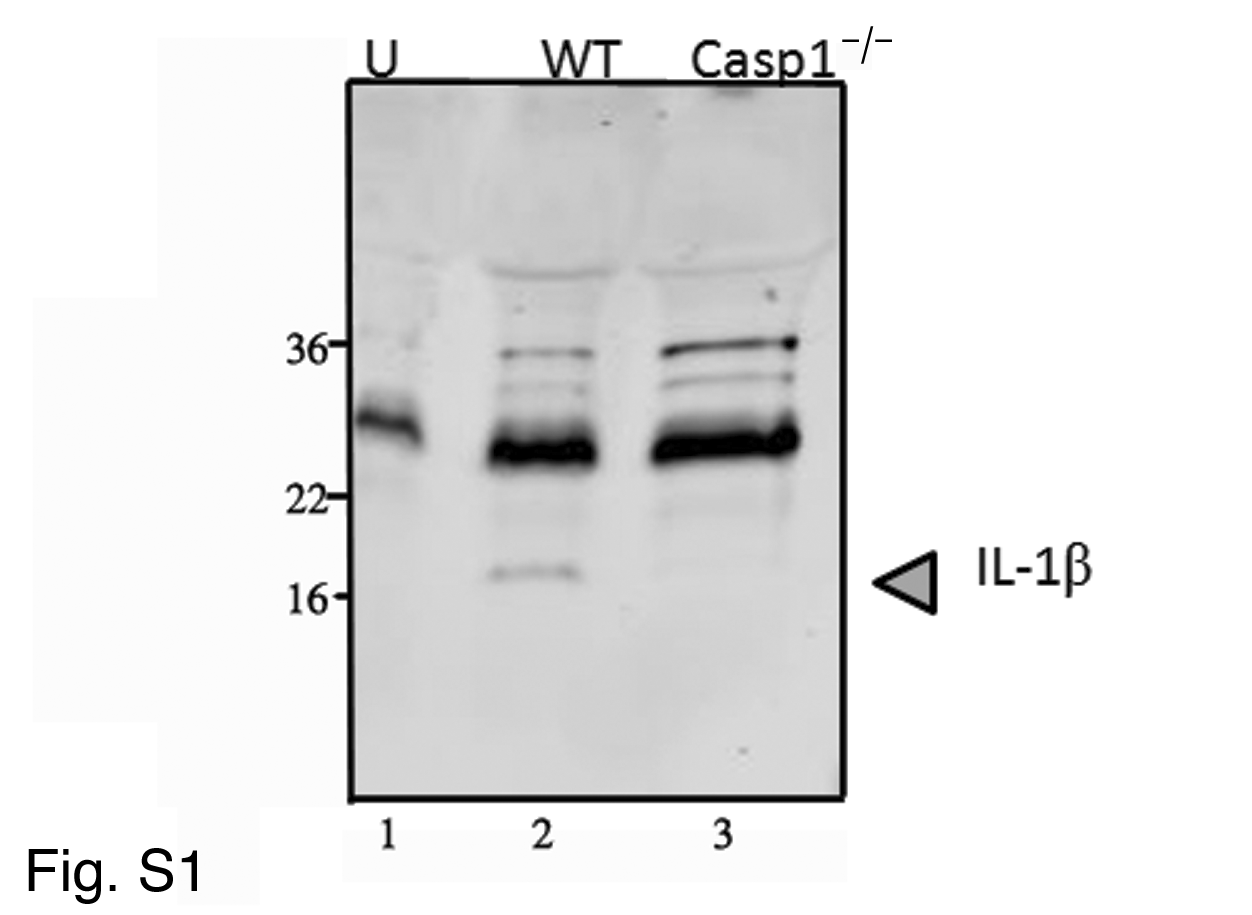
**

**Figure S5. Detection of processed IL-1β in supernatants of wild-type or *casp-1*^-/-^ macrophages infected with Yp-YopJ^KIM^.**  Wild-type macrophages were left uninfected (U) or infected with Yp-YopJ^KIM^ (WT), and in parallel *casp-1*^-/-^macrophages were infected with Yp-YopJ^KIM^ (Casp1^-/-^). At 24 hr post infection, supernatants were collected and immunoprecipitated with anti-IL-1β antibody and protein G beads. Samples of the immune complexes were processed for SDS-PAGE and immunoblotting. Immunoblotting was performed with anti-IL-1β antibody. Positions of molecular weight standards (kDa) are shown on the left. The position of cleaved IL-1β at 17 kDa is indicated on the right.


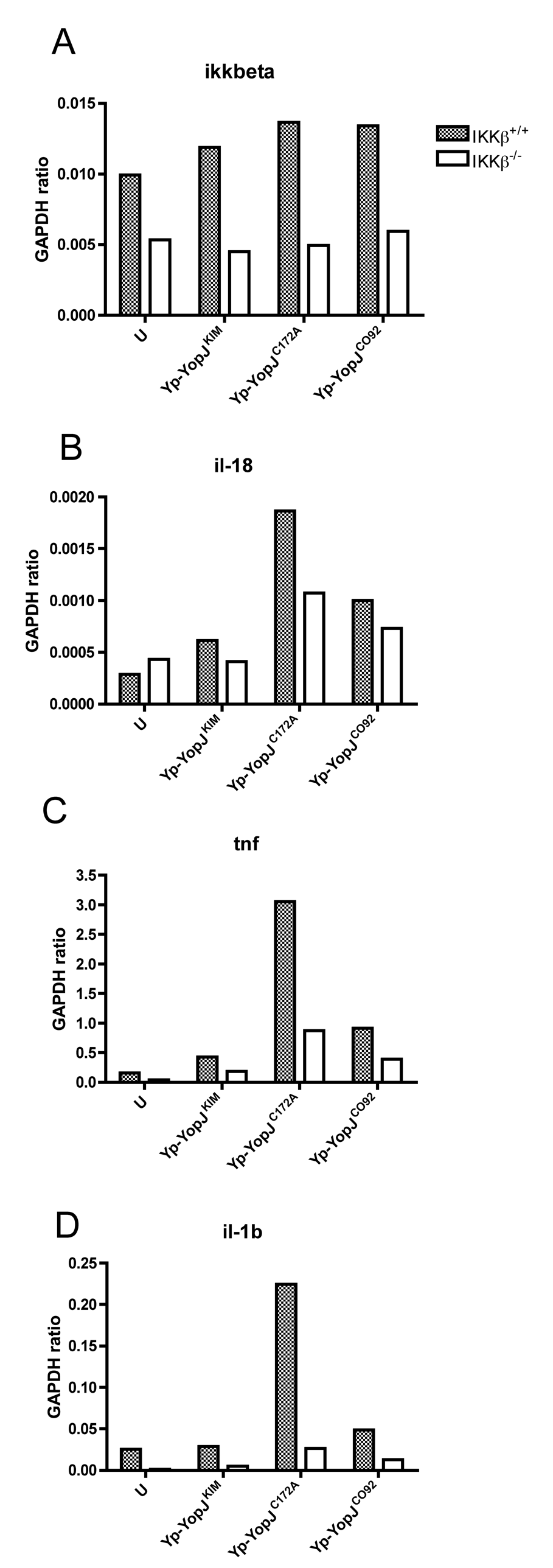


**Figure S6. Measurement of *Ikk***β **and cytokine message levels by quantitative real time PCR.** *Ikk*β^F/F^ or *Ikk*β^Δ^ macrophages from the same batch of cells analyzed in Fig. 4 and Fig. 5 of the paper were infected with the indicated Yp-YopJ strains at an MOI of 50. At 4 hr post infection, mRNA was extracted and analyzed by qRT-PCR to quantify levels of ikkbeta (A), il-18 (B), tnf (C) or il-1b (D). Results are from one experiment.

**
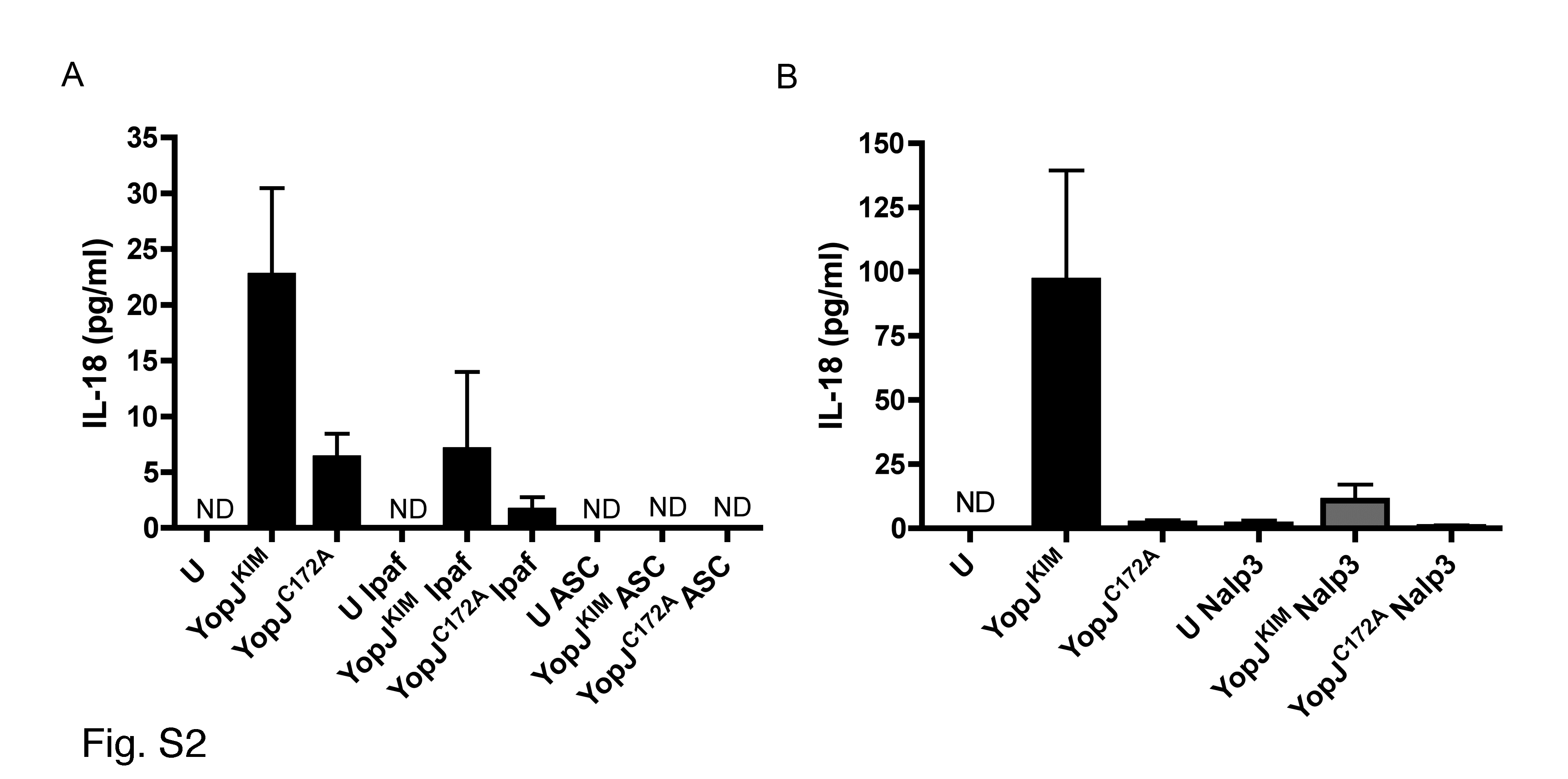
**

**Figure S7. Determination of the importance of inflammasome components for the secretion of IL-18 during Yp-YopJ^KIM^ infection.** Wild-type, NLRC4 (Ipaf)-, ASC (ASC)- or NLRP3 (Nalp3)-deficient BMDMs were left uninfected (U) or infected with Yp-YopJ^KIM^ or Yp-YopJ^C172A^ at an MOI of 10. Supernatants were collected at 24 hr post-infection and analyzed to determine amounts of secreted IL-18 by ELISA. Results shown in panels A and B are the average of three independent experiments. Error bars represent standard deviation. N.D., not detected.

**Supplemental Experimental Procedures**

**IL-1β immunoprecipitation assay.** Supernatants of uninfected wild-type BMDMs, wild-type BMDMs infected with with Yp-YopJ^KIM^, or *casp-1^-/-^* macrophages infected with with Yp-YopJ^KIM^ (MOI of 10) were collected at 24 hr post infection. One ml of supernatant from each sample was centrifuged and transferred to a new tube. One microgram of mouse monoclonal anti-IL-1β antibody (R&D Systems) and 10 μl of pre-washed Protein G Sepharose 4B beads (Sigma) were added and tubes were rotated at 4°C overnight. The next day, samples were centrifuged at 4°C for 5 minutes and the supernatant was discarded. Beads were washed twice in PBS and boiled in 40 μl of 1× Laemmli sample buffer. Samples were processed for immunoblotting using the same anti-IL-1β antibody used for immunoprecipitation. IRDye800 conjugated goat anti-mouse antibody (Rockland) was used as the secondary antibody. The blots were scanned by Odyssey (LI-COR).

**Immunoblotting of *Y. pestis* lysates**. *Y. pestis* strains were grown in defined TMH medium at 28°C overnight. Cultures were diluted 1 to 20 in TMH containing 2.5mM Ca^2+^, shaken at 28°C for 2 hours and transferred to 37°C for 4 hours with shaking to induce Yop expression. Bacteria were normalized by optical density (OD) and spun down. Pellets were boiled in 1× Laemmli buffer. Samples of the lysates were resolved by 10% SDS-PAGE and processed for immunoblotting using mouse anti-YopJ monoclonal antibody [2] at a 1:1000 dilution at 4°C overnight. IRDye800 conjugated goat anti-mouse antibody (Rockland) was used as the secondary antibody. The blots were scanned by Odyssey (LI-COR) and reprobed using a mouse monoclonal anti-YopH antibody as the loading control.

**Immunoblotting of secreted Yops.** IP26 strains containing pBAD plasmids encoding different YopJ isoforms were grown overnight with shaking in LB broth at 28°C. The next day, the cultures were diluted 1:40 in LB containing 20 mM NaOX and 20 mM MgCl_2_ and incubated at 28°C with shaking for 2 hr. To induce Yop expression, 0.2% arabinose was added to cultures and incubation was continued for 4 hr at 37°C with aeration. Bacteria were normalized by OD_600_ measurements and centrifuged to collect supernatants. Trichloroacetic acid (TCA) was added to supernatants to a final concentration of 10% and samples were rotated overnight at 4°C. The next day, samples were centrifuged at maximum speed in a microcentrifuge for 30 minutes at 4°C and supernatants were discarded. Pellets were washed with cold acetone and centrifuged at maximum speed for 5 minutes at 4°C after which the acetone was discarded and samples were dried by vacuum centrifugation. Pellets were dissolved in 1X Laemmli buffer, and the resulting samples were resolved by SDS-PAGE (10% gels) and processed for immunoblotting. Immunoblots were developed with mouse anti-GSK monoclonal antibody (Cell Signaling) and goat anti-mouse IRDye800 secondary antibody (Rockland). The blots were scanned by Odyssey (LI-COR). Immunoblots were reprobed with polyclonal rabbit anti-YopE antibody [3] as a loading control.

**qRT-PCR.** Infections of BMDMs were carried out with 1x10^6^ BMDMs infected at an MOI of 50. At 4 hr post infection, RNA extraction, reverse transcription and real-time quantitative PCR were done as described before [4]. The sequences of primer pairs used were: for il-1b 5'- TACAAGGAGAACCAAGCAACGAC -3' and 5'- GCCCATACTTTAGGAAGACACGG -3'; for il-18 5’-GCGTCAACTTCAAGGAAATGATG -3' and 5'- TCACAGAGAGGGTCACAGCCAGTCC -3’. The primers used for ikkbeta were 5'- GTGGAGCCTGGGAAATGAAAG -3' and 5'- TAAGAGCCGATGCGATGTCAC -3'. The primer pairs used for GAPDH and tnf were as described previously [4].

**Supplemental References**

1. Lilo S, Zheng Y, Bliska JB (2008) Caspase-1 activation in macrophages infected with Yersinia pestis KIM requires the type III secretion system effector YopJ. Infect Immun 76: 3911-3923.

2. Zhang Y, Murtha J, Roberts MA, Siegel RM, Bliska JB (2008) Type III secretion decreases bacterial and host survival following phagocytosis of Yersinia pseudotuberculosis by macrophages. Infect Immun 76: 4299-4310.

3. Black DS, Bliska JB (2000) The RhoGAP activity of the *Yersinia pseudotuberculosis* cytotoxin YopE is required for antiphagocytic function and virulence. Mol Microbiol 37: 515-527.

4. Zhang Y, Ting AT, Marcu KB, Bliska JB (2005) Inhibition of MAPK and NF-kappa B pathways is necessary for rapid apoptosis in macrophages infected with *Yersinia*. J Immunol 174: 7939-7949.
